# Calcium Channel Blockers Inhibit Pancreatic Neuroendocrine Neoplasms Progression via Cav1.2‐Epigenetic Circuit

**DOI:** 10.1002/advs.202516733

**Published:** 2026-02-04

**Authors:** Yangyinhui Yu, Qiongcong Xu, Jinzhao Xie, Mingjian Ma, Xitai Huang, Yinhao Shi, Jiawei Zhou, Enliang Zhu, Ziyi Zhao, Ning Zhang, Zhide Liu, Jingyuan Ye, Xiaoyu Yin

**Affiliations:** ^1^ Department of Pancreato‐Biliary Surgery the First Affiliated Hospital of Sun Yat‐Sen University Guangzhou China; ^2^ Department of Gastroenterology & Hepatology the First Affiliated Hospital of Sun Yat‐Sen University Guangzhou China

**Keywords:** calcium channel blockers, calcium signaling, epigenetics, pancreatic neuroendocrine neoplasms

## Abstract

Pancreatic neuroendocrine neoplasms (pNEN) are rarely encountered, accounting for about 2% of all pancreatic neoplasms. Disease progression is frequently observed as recurrence or distal metastasis. Mechanisms underlying pNEN progression are still poorly investigated, and treatments against pNEN are challenging due to the pronounced neoplastic heterogeneity. Here, by performing clinicomolecular analysis, we report a novel mechanism of positive regulatory circuit between Cav1.2‐mediated calcium signaling and epigenetic control by H3K27 acetylation (H3K27ac). Tumor‐cell‐specific expression of Cav1.2 strongly contributes to disease progression and correlates with malignant biological behaviors of pNEN. Moreover, we find calcium channel blockers (CCBs), especially amlodipine, remarkably inhibit pNEN progression in vitro and in vivo. Clinically, administration of CCBs correlates with better progression‐free survival (PFS) and a lower rate of distal metastasis. Our work uncovers the novel mechanism of the Cav1.2‐epigenetic circuit and expands the scope of therapeutic strategy for further investigation in pNEN.

## Graphical abstract

Our study reveals a novel mechanism of a positive regulatory circuit between Cav1.2 and H3K27ac for pancreatic neuroendocrine neoplasms (pNENs) progression. Cav1.2 is identified as a crucial target for promoting disease progression and correlates with malignant behaviors, which are remarkably inhibited by the administration of calcium channel blockers (CCBs). This study provides a novel therapeutic target and strategy against pNEN progression.

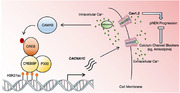

## 1 Introduction

Pancreatic neuroendocrine neoplasms (pNEN) are rare and heterogeneous diseases originating from pancreatic neuroendocrine cells. The reported incidence of pNEN is about 1 case per 100 000 individuals and accounts for about 2% of all types of pancreatic neoplasms [1, 2]. Due to improvements in diagnostic technologies, like Gallium (^68^Ga)‐based Positron Emission Tomography/Computed Tomography (PET/CT), the incidence of pNEN has risen multiple times over the past few decades [3].

According to the World Health Organization (WHO) classification of gastroenteropancreatic neuroendocrine neoplasms, pNEN is sub‐classified as well‐differentiated pancreatic neuroendocrine tumor (pNET) and poorly differentiated pancreatic neuroendocrine carcinoma (pNEC), based on the pathological evaluation of proliferation index Ki‐67 and mitoses [4]. Although several neuroendocrine immunohistochemical markers, such as chromogranin A (CgA) and synaptophysin (SYP), are commonly expressed in pNEN [5], the biological behavior of which is of dramatic discrepancy due to the neoplastic heterogeneity. Such variability, including the ability of hormone secretion and distal metastasis, potential for malignant transformation, as well as molecular abnormalities, is prominent not only among different individuals, but also different sites within neoplasms [6, 7]. Treatment against pNEN remains challenging due to such neoplastic heterogeneity. Surgery is recommended only when the pNET tumor size is over 2 cm with no hormone secretion or any size with hormone secretion [4]. A newly introduced option, peptide receptor radionuclide therapy (PRRT), is clinically unsubstantiated and still under exploration [8]. Somatostatin analogues (SSAs)‐based targeted therapy, which activates somatostatin receptors (SSTRs), such as octreotide or lanreotide, is the most commonly used agent for anti‐proliferation and symptoms relieving by patients with low‐grade pNET [9, 10, 11]. However, limited efficacy and endocrine‐related side effects make it unsatisfactory for disease control in the long term [12].

The human genome is compacted in chromatin in cellulo, and genes encoded are regulated by active cis‐regulatory elements, which require histone modifications, such as H3K27ac, H3K4me3, and other covalent markers. H3K27ac marks enhancers and super‐enhancers for gene activation and tumor cell functioning [13, 14, 15]. Previously reported mechanisms on pathogenesis and progression of pNEN are diverse, including *MEN1* mutation, alternative lengthening of telomeres (ALT) associated with loss of *ATRX/DAXX*, and aberrant signaling on PI3K/Akt/mTOR [16]. It is recently reported that epigenetic marks of H3K27ac play a critical role in regulating key transcriptional factors *ARX* and *PDX1*, thus correlating with post‐operative relapse, stressing the importance of epigenetic control for H3K27ac in pNEN [17]. However, the detailed mechanism of H3K27ac regulation and its contribution to pNEN progression is still less known.

Calcium signaling is highly conserved among species and was first discovered as a key regulator for myocardial and neuronal excitation [18]. Several molecules participate in this process, including calcium channels, pumps, and exchangers. As the most important one for the calcium channels, it controls the influx of extracellular calcium, which may exceed the concentration of intracellular calcium by more than 1 mm, a thousand times more than before opening [18, 19]. Several recent studies have uncovered the role of calcium signaling in cancer biology [20], but haven't in pNEN. Calcium channel blockers (CCBs), a series of widely used drugs, are capable of blocking the different types of calcium channels and are frequently prescribed to patients with cardiovascular and cerebrovascular diseases [21, 22]. However, whether calcium signaling participates in pNEN progression and whether CCBs are effective against pNEN progression have not been established.

In this study, we performed clinicomolecular analysis of pNEN, identifying Cav1.2 as the critical target, which significantly promotes pNEN progression. Mechanistically, Cav1.2 and H3K27ac function as a positive regulatory circuit via the CaM‐CAMKII‐CREB1 pathway. From a therapeutic perspective, we found CCBs, a well‐known Cav1.2 channel blocker, especially amlodipine, are strongly capable of inhibiting pNEN progression via both experimental and clinical aspects. Together, this study strengthens the pivotal circuit between epigenetic control of H3K27ac with calcium signaling and identifies Cav1.2 as the key role in promoting pNEN progression. Moreover, it is found that administration of CCBs correlates with improved progression‐free survival and lower rates of distal metastasis. Based on these findings, we propose a new therapeutic strategy that expands the scope of treatment against pNEN.

## 2 Results

### 2.1 Aberrant H3K27 Acetylation Mediated by P300/CBP Associates With Poor Prognosis and Regulates Calcium Signaling Pathway in pNEN

**FIGURE 1:**
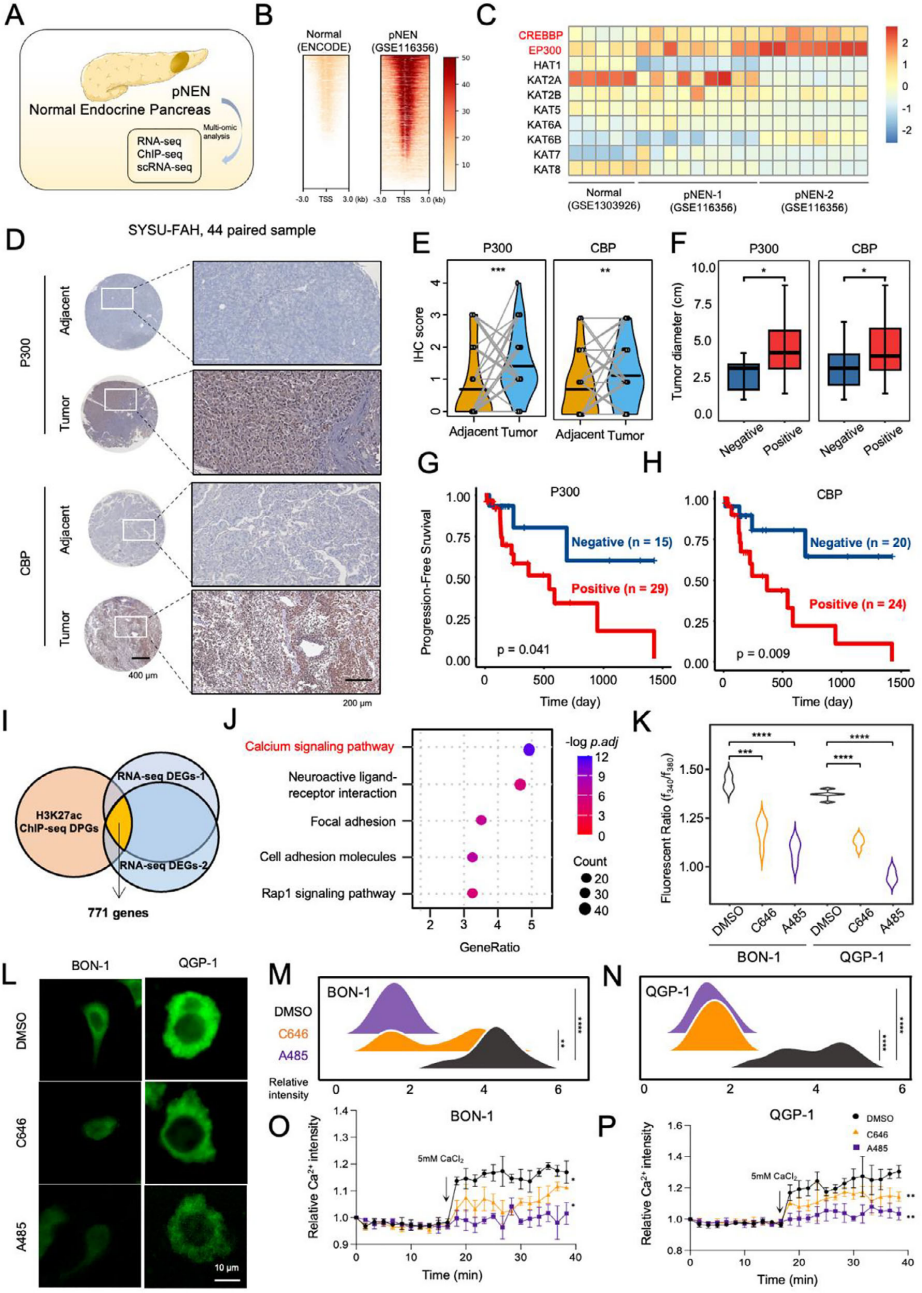
Aberrant H3K27ac mediated by P300/CBP associates with poor prognosis and regulates calcium signaling activation in pNEN. (A) Schematic showing the multi‐omic analysis of pNEN and normal endocrine pancreas. (B) Public datasets (data from ENCODE and GSE116356) reveal global H3K27ac signals enriched in promoter regions (3 kb upstream and downstream of the transcription start site). (C) Heatmap showing the expression of histone acetylases among pNEN and normal endocrine pancreas according to public datasets (GSE1303926 and GSE116356). *EP300* and *CREBBP* were the most remarkably up‐regulated genes, suggesting their key role in regulating H3K27 acetylation in pNEN. (D) Representative images of IHC staining of P300 and CBP in paired normal and pNEN tissue microarrays. Scale bars: 400 and 200 µm. (E) Statistics of IHC score for P300 and CBP, higher expression levels of P300 and CBP were found in pNEN than in paired adjacent normal tissues. ^**^ *p* < 0.01; ^***^ *p* < 0.001 according to paired Student's t‐test. Data were shown as mean ± SD. Sample size *n* = 44 pairs. (F) Boxplot of tumor diameter between the negative and positive groups based on the differential IHC score of P300 or CBP. ^*^ *p* < 0.05 according to unpaired two‐tailed Student's t‐test. The box represents the interquartile range; whiskers are 1.5× the interquartile range. (G, H) Kaplan‐Meler analysis of the PFS rate of pNEN patients among the two groups. *P*‐value was calculated by the Log‐rank test. (I) Venn diagram showing the overlapped genes between two up‐regulated up‐DEGs and H3K27ac DPGs. There were 771 overlapped genes in total. (J) KEGG enrichment analysis revealed the significant activation of the calcium signaling pathway in pNEN. (K) Intracellular calcium concentration measured in BON‐1 and QGP‐1 cells. A485 and C646 significantly lowered intracellular calcium. ^***^ *p* < 0.001; ^****^ *p* < 0.0001 according to one‐way ANOVA with Tukey's multiple comparisons tests. Sample size *n* = 5. (L) Calcium imaging of GCaMP6s in BON‐1 and QGP‐1 cells. A485 and C646 lowered the cytosolic calcium level. Scale bar: 10 µm. Sample size *n* = 3. (M, N) Quantification of cytosolic calcium decrease on A485 or C646 treatment in BON‐1 (M) and QGP‐1 (N). ^**^ *p* < 0.01; ^***^ *p* < 0.001; ^****^ *p* < 0.0001 according to one‐way ANOVA with Tukey's multiple comparisons tests. (O, P) Extracellular calcium flux assay. 5 mm CaCl_2_ was added as extracellular calcium. Influx of calcium was partially blocked after A485 and C646 treatment. ^*^ *p* < 0.05; ^**^ *p* < 0.01 according to unpaired two‐tailed Student's t‐test. Data were shown as mean ± SD.

To explore the epigenetic regulation network in pNEN progression, we conducted multi‐omic analysis in a public database between pancreatic neuroendocrine neoplasms (pNEN) and normal endocrine pancreas (Figure 1A; Figure S1A). First, we analyzed the global H3K27 acetylation (H3K27ac) profile. By exploring H3K27ac chromatin immunoprecipitation followed by sequencing (ChIP‐seq) data, it was found that the global H3K27ac signal at promoter regions was dramatically enhanced in pNEN tissue compared with the normal endocrine pancreas (Figure 1B), indicating the pivotal role of H3K27ac in pNEN.

Since the epigenetic regulation of H3K27ac was mediated by several modification enzymes, we then explored the gene expression of candidate enzymes, and it was found that *CREBBP* (CBP) and *EP300* (P300) were significantly upregulated in pNEN (Figure 1C). To further assess the role of P300/CBP, we explored the difference in expression in 44 paired sample tissue microarrays (TMA) via immunohistochemistry (IHC) and found the elevated IHC score in the tumor compared with the adjacent normal (Figure 1D,E). Next, we divided these patients into negative or positive groups according to the differential expression of the tumor by adjacent normal tissue. Combined analysis with clinical data revealed a larger tumor diameter in the positive group than in the negative group (Figure 1F). Moreover, we observed better progression‐free survival (PFS) in the positive group compared with the negative group (Figure 1G,H). These data suggested that P300/CBP might be the key enzymes for H3K27ac modification in pNEN and were strongly associated with poor prognosis, indicating the significance of P300/CBP and H3K27ac in pNEN progression.

Next, we included transcriptomic profiles by RNA‐seq from two datasets for integrated analysis. By combining H3K27ac targeting differential peaks related genes (DPGs) from H3K27ac ChIP‐seq and up‐regulated differentially expressed genes (DEGs) from RNA‐seq, there were 771 genes identified (Figure 1I). Kyoto Encyclopedia of Genes and Genomes (KEGG) enrichment analysis was first processed, showing that the most significantly enriched pathway was the calcium signaling pathway (Figure 1J), and gene ontology (GO) analysis also indicated the corresponding biological process, such as calcium ion transport (Figure S1B). Gene Set Enrichment Analysis (GSEA) analysis also illustrated the significance of enrichment for the calcium signaling pathway in pNEN (Figure S1C). Moreover, ligand‐receptor analysis revealed the most significant role of calcium in pNEN (Figure S1D). Moreover, we analyzed the genome‐wide distribution of differential peaks of H3K27ac and expression status of calcium signaling pathway‐related genes, to see whether the regulation‐activation pattern exists in the above genes. The results showed that differential peaks of H3K27ac were enriched in those gene loci representing the calcium signaling pathway, compared with genome‐wide distribution (Figure S1E). Moreover, the expression of calcium signaling pathway‐related genes was higher in the two pNEN group comparing with the normal group (Figure S1F).

Further, we explored the regulatory role of H3K27ac toward calcium signaling. To modify the cellular H3K27ac states by enzymatic inhibition, we applied two small molecular inhibitors, A485 and C646, which were previously reported to function as the dual inhibitor of P300/CBP and a selective P300 inhibitor, which was used to interfere with H3K27ac modification (Figure S1G). As expected, two inhibitors lowered the H3K27ac level in two pNEN cell lines, BON‐1 and QGP‐1 (Figure S1H). By measuring the intracellular calcium concentration in BON‐1 and QGP‐1 between the control (DMSO) and treatment (A485 and C646) groups, we found that endogenous calcium concentration was significantly lower than that of the control group (Figure 1K). To deeply investigate the intracellular calcium level, we applied calcium imaging with GCaMP6s, a widely used calcium indicator [23]. Similarly, after transfection into BON‐1 and QGP‐1 cells, we found that the fluorescent intensity of GCaMP6s was significantly lower in the A485 or C646 treatment group (Figure 1L–N). Moreover, when adding the exogenous calcium (5 mm CaCl_2_) to the culture media, the intracellular calcium concentration did not elevate (Figure 1K,L), which suggested that inhibitor treatment may induce silenced expression of calcium signaling effectors.

Taken together, these results clarify that the aberrant H3K27ac mediated by P300/CBP activates calcium signaling in pNEN, indicating that they may play a key role in pNEN progression.

### 2.2 pNEN‐Specific Expression of Cav1.2 Aggregates upon Ca^2+^ Stimulation

**FIGURE 2:**
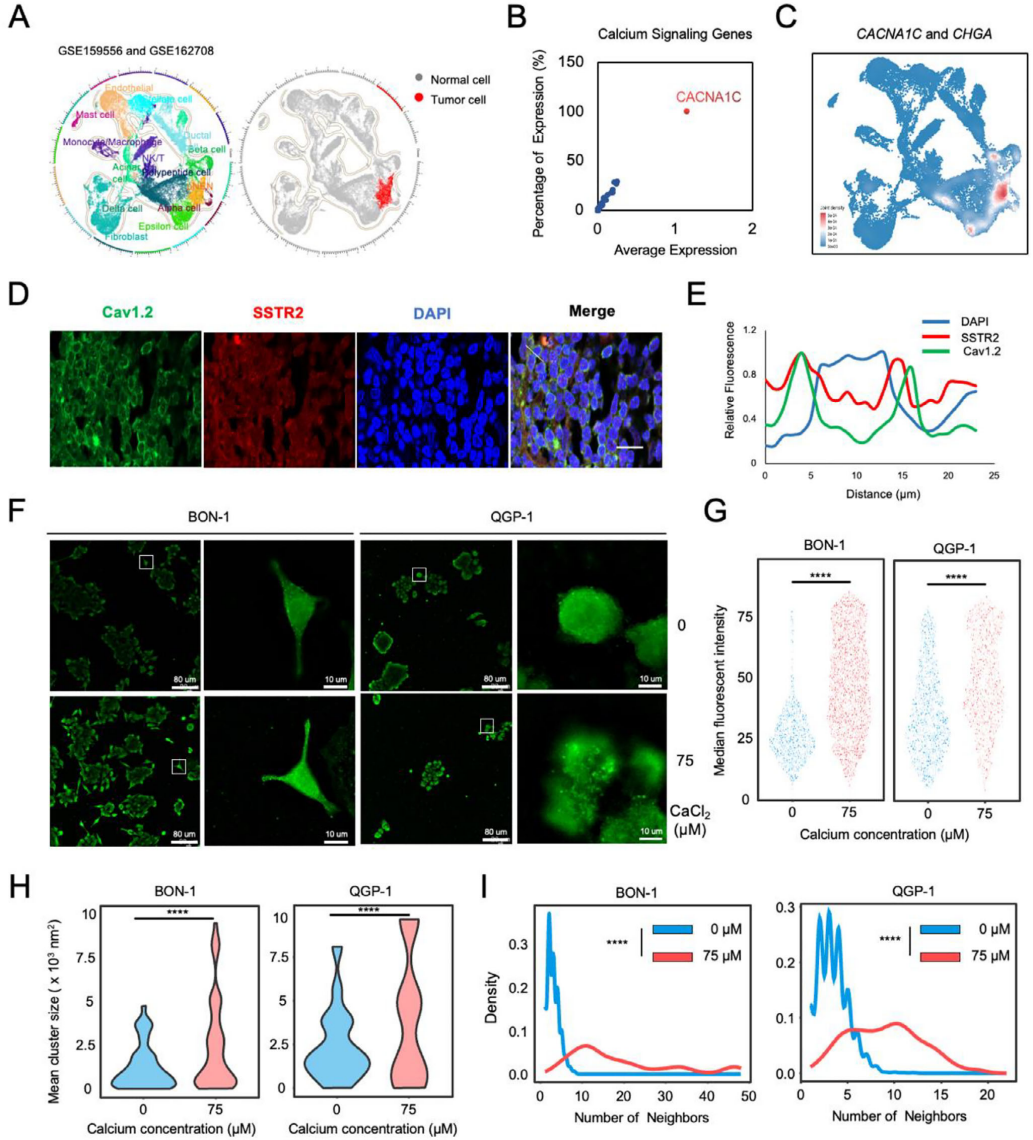
pNEN‐specific expression of Cav1.2 aggregates upon Ca^2+^ stimulation. (A) Projection onto two dimensions of all cells by UMAP showing different cell types of single‐cell transcriptomics using public datasets (data from GSE159556 and GSE162708). All cell types (left) and tumor cells (right) were shown. (B) Specificity analysis among all genes in the calcium signaling pathway. *CACNA1C* was the most prominent gene, indicating the key role of *CACNA1C* with specific expression. (C) Joint density plot showing co‐expression of *CACNA1C* with the representative neuroendocrine marker genes *CHGA*. (D) Representative images of immunofluorescence showing co‐localization of Cav1.2 and SSTR2. Scale bar: 30 µm. Sample size *n* = 5. (E) Fluorescent line profile of Cav1.2 (green), SSTR2 (red), and DAPI (blue) signal. (F) Representative images of Cav1.2 fluorescence by TIRF imaging with or without exogenous calcium. Scale bar: 80 µm for lower magnification and 10 µm for higher magnification. Sample size *n* = 3. (G) Quantification of the median fluorescent intensity of Cav1.2 per cell. ^****^ *p* < 0.0001 according to unpaired two‐tailed Student's t‐test. (H) Aggregation analysis of the mean cluster size of Cav1.2 in BON‐1 and QGP‐1 cells. ^****^ *p* < 0.0001 according to unpaired two‐tailed Student's t‐test. (I) Density distribution of the number of Cav1.2 puncta neighbors. Significance was assessed with the Mann‐Whitney U test.

To identify the key genes of the calcium signaling pathway in single cell level, we next enrolled single‐cell transcriptomic analysis between pNEN and the normal endocrine pancreas. Public datasets of scRNA‐seq, of which 30 781 cells in total, were integrated for comparative analysis, annotated into 14 cell subtypes, and visualized using a uniform manifold approximation and projection (UMAP)‐based algorithm (Figure 2A). The proportion of each cell type varies between the normal endocrine pancreas and pNEN group (Figure S2A). By spearman correlation test for the top 1000 genes with large standard deviation, it was shown that endocrine or exocrine‐related clusters, immune clusters, and mesenchymal clusters were distinctive (Figure S2B). For mesenchymal clusters, fibroblasts (*n* = 5747, *COL3A1*), endothelial cells (*n* = 3231, *PECAM1*), and stellate cells (*n* = 1876, *CPXM2*) were identified. Immune clusters like NK/T cells (*n* = 1470, *CD3D* and *CD3E*), monocyte/macrophages (*n* = 985, *CD14*), and mast cells (*n* = 367, *TPSB2* and *TPSAB1*) were also identified, which were consistent with the immune infiltration profiles in pNEN as previously reported [24]. Exocrine‐related clusters, in which pancreatic acinar cells (*n* = 2236, *CPA2*) and ductal cells (*n* = 2961, *KRT19*) were identified in this dataset. For endocrine clusters, alpha cells (*n* = 373, *GCG* and *PLCE1*), beta cells (*n* = 1672, *PDX1*), delta cells (*n* = 5418, *SST*), polypeptide cells (*n* = 818, *PPY*), and newly identified epsilon cells (*n* = 2182, *GHRL*) were distinguished by their endocrine bioactive characteristics [25], respectively (Figure S2C). Notably, *SSTR2*‐expressing subtype (*n* = 1445) was also identified and considered as the tumor cells based on the comprehensive knowledge of onco‐biological roles of pNEN [26]. To further validate the neoplastic identity of this subtype, we performed Copykat analysis, which was applied for tumor cell identification based on the copy number variations (CNVs) (Figure S2D). As expected, the aneuploid cells were almost of complete accordance (Figure 2A). These results indicate that tumor cells were identified with prominent CNVs, which were consistent with their molecular characteristics [27].

Next, we aimed to find the pNEN‐specific expression genes in the calcium signaling pathway. There were 36 genes in total that were previously identified by KEGG analysis; they were subsequently ranked by their average expression and percentage of expression in 1445 pNEN cells. It was noticed that *CACNA1C* (Voltage‐dependent L‐type calcium channel subunit alpha‐1C, the most important subunit of Cav1.2 channel), whose score was extraordinarily higher than that of other candidates (Figure 2B; Figure S2E). It was previously reported that *CACNA1C* is expressed on neurons of the central nervous system (CNS) or excitation transmission cells in cardiac tissue [28, 29], but not in pNEN so far. Further analysis showed that *CACNA1C* was expressed neither in normal endocrine pancreas nor in non‐tumor cells in pNEN (Figure S2F,G). Moreover, we analyzed the co‐expression pattern of *CACNA1C* and neuroendocrine markers *CHGA* (CgA), and results showed a high density of joint expression (Figure 2C), indicating that cells with high‐level expression of *CACNA1C* correlated with predominant neuroendocrine behaviors. To make further validation, we collected the slides from pNEN samples and tested the cell‐type‐specific expression of Cav1.2 by immunofluorescence (IF). We found that Cav1.2 is located in the cell membrane and co‐expressed with SSTR2 (Figure 2D), which was considered the canonical marker of pNEN. Signals of Cav1.2 and SSTR2 were subsequently analyzed, and we found that two signals were of co‐occurrence (Figure 2E; Figure S2H). Collectively, we conclude that *CACNA1C* is specifically expressed in pNEN cells.

Furthermore, we investigated the functional characteristics of the Cav1.2 channel responsible for cellular calcium signaling activation. By performing Total Internal Reflection Fluorescence (TIRF) microscopy to track Cav1.2 at single molecular level. We discovered that there were more puncta of Cav1.2 signal with exogenous calcium stimulation (Figure 2F). These puncta showed elevated median fluorescent intensity (Figure 2G), indicating that Cav1.2 aggregation to form clusters, which was consistent with previous studies [30]. Next, we analyzed the size of these clusters; as expected, the mean cluster size was larger when adding exogenous calcium, both in BON‐1 and QGP‐1 cells (Figure 2H). Moreover, neighboring cluster analysis (defined as the number of clusters proximal to a certain cluster) showed that, with exogenous calcium, the differential density distribution of neighbors indicated the tendency of aggregation upon calcium stimulation (Figure 2I). These data demonstrated that Cav1.2 aggregates in response to calcium stimulation, promoting intracellular calcium signaling activation.

### 2.3 Cav1.2 Promotes pNEN Progression

**FIGURE 3:**
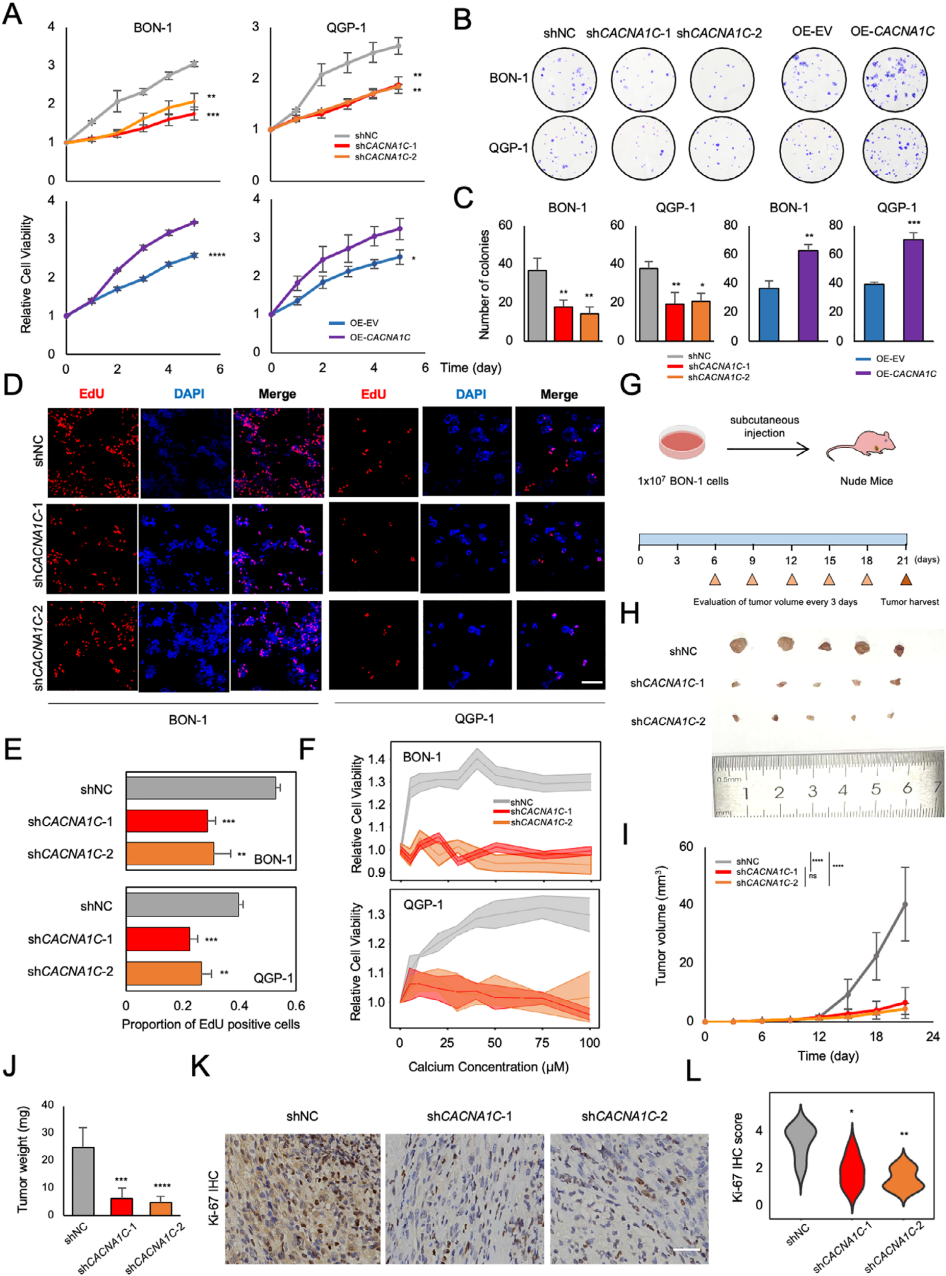
Cav1.2 promotes pNEN progression. (A) CCK‐8 assay depicting in vitro growth rate of control and stable knockdown or over‐expression of CACNA1C in BON‐1 and QGP‐1 cells. ^*^ *p* < 0.05; ^**^ *p* < 0.01; ^***^ *p* < 0.001; ^****^ *p* < 0.0001 according to one‐way ANOVA with Tukey's multiple comparisons tests (shNC, sh*CACNA1C*‐1 and sh*CACNA1C*‐2) or unpaired two‐tailed Student's t‐test (OE‐EV and OE‐*CACNA1C*). Data were shown as mean ± SD. (B, C) Representative images of colonies formed by control and stable knockdown or over‐expression of *CACNA1C* in BON‐1 and QGP‐1 cells (B) and quantification statistics (C). ^*^ *p* < 0.05; ^**^ *p* < 0.01; ^***^ *p* < 0.001; ^****^ *p* < 0.0001 according to one‐way ANOVA with Tukey's multiple comparisons tests (shNC, sh*CACNA1C*‐1 and sh*CACNA1C*‐2) or unpaired two‐tailed Student's t‐test (OE‐EV and OE‐*CACNA1C*). Data were shown as mean ± SD. (D, E) Representative images of EdU staining among the negative control or stable knockdown group in BON‐1 and QGP‐1 cells (D) and proportion analysis of EdU‐positive cells per field (E). Scale bar: 100 µm. ^*^ *p* < 0.05; ^**^ *p* < 0.01; ^***^ *p* < 0.001; ^****^ *p* < 0.0001 according to one‐way ANOVA with Tukey's multiple comparisons tests. Data were shown as mean ± SD. Sample size *n* = 3. (F) Calcium‐responsive viability in BON‐1 and QGP‐1 cells upon different exogenous calcium incubation between the negative control and the two stably knockdown groups. Data were shown as mean ± SD. (G) Schematic showing the process of BON‐1 cell‐derived xenografts establishment. (H) Macroscopic presentation of BON‐1 CDXs among both control and two independent stable knockdown groups. (I) Tumor volume was calculated every three days post‐inoculation of BON‐1 cells.ns (not significant); ^****^ *p* < 0.0001 according to one‐way ANOVA with Tukey's multiple comparisons tests. Data were shown as mean ± SD. Sample size *n* = 5. (J) Tumor weight of the last day was measured in control and stable knockdown of *CACNA1C* group. ^***^ *p* < 0.001; ^****^ *p* < 0.0001 according to one‐way ANOVA with Tukey's multiple comparisons tests. Data were shown as mean ± SD. Sample size *n* = 5. (K) Representative images of IHC staining of the Ki‐67 for CDXs among the negative control and knockdown groups. Scale bar: 20 µm. Sample size *n* = 5. (L) Quantification of Ki‐67 IHC score CDXs. ^*^ *p* < 0.05; ^**^ *p* < 0.01 according to one‐way ANOVA with Tukey's multiple comparisons tests. Sample size *n* = 5.

To elucidate the role of Cav1.2 in pNEN progression, we first generated two independent knockdowns and one overexpression of *CACNA1C* in both BON‐1 and QGP‐1 cell lines, since *CACNA1C* was considered the most significant subunit of Cav1.2. Western blot and qRT‐PCR were performed to validate the interference effectiveness in protein (Figure S3A,B) and mRNA level (Figure S3C,D). Next, we tested whether *CACNA1C* contributed to pNEN progression by several functional assays. In vitro proliferation assay showed the retarded cell growth rate of knockdown of *CACNA1C*, while enhanced growth rate of over‐expression of *CACNA1C* was observed for both cell lines (Figure 3A). Colony formation assay also showed the reduced number of clones in knockdown of *CACNA1C* and increased number in over‐expression of *CACNA1C* (Figure 3B,C). We also performed a click chemistry‐based assay using 5‐ethynyl‐2’‐deoxyuridine (EdU) to evaluate the cell proliferation state. The proportion of EdU‐positive cells per field was significantly lower in both stably knockdown of *CACNA1C* groups (Figure 3D,E), indicating the pivotal role of Cav1.2 for pNEN progression.

Next, we performed calcium‐responsive viability to elucidate the synergistic role of Cav1.2 with calcium for cellular proliferation. By adding different concentrations of exogenous calcium, cells with *CACNA1C* knockdown showed no response to exogenous calcium stimulation, while the control group showed a concentration‐dependent growth pattern (Figure 3F). These data indicated that Cav1.2 is required for over‐activated calcium signaling to control cellular proliferation.

Furthermore, we generated BON‐1 cell‐derived xenografts (CDXs) in nude mice to make in vivo validation (Figure 3G). By comparing with two knockdown groups with the control group, we found the tumor size was significantly lowered (Figure 3H), both tumor volume and tumor weight were less in the knockdown group than in the control group (Figure 3I,J), at meantime the body weight of mice in each group were consistent (Figure S3E). To further evaluate the proliferation properties of CDXs, we performed IHC staining of Ki‐67, and the results showed decreased expression of Ki‐67 level in the knockdown group compared with the control group (Figure 3K,L), which was consistent with the results of the in vitro proliferation assay. Taken together, these data strongly indicated that Cav1.2 promotes pNEN progression.

### 2.4 Cav1.2 Correlates with Malignant Biological Behaviors of pNEN

**FIGURE 4:**
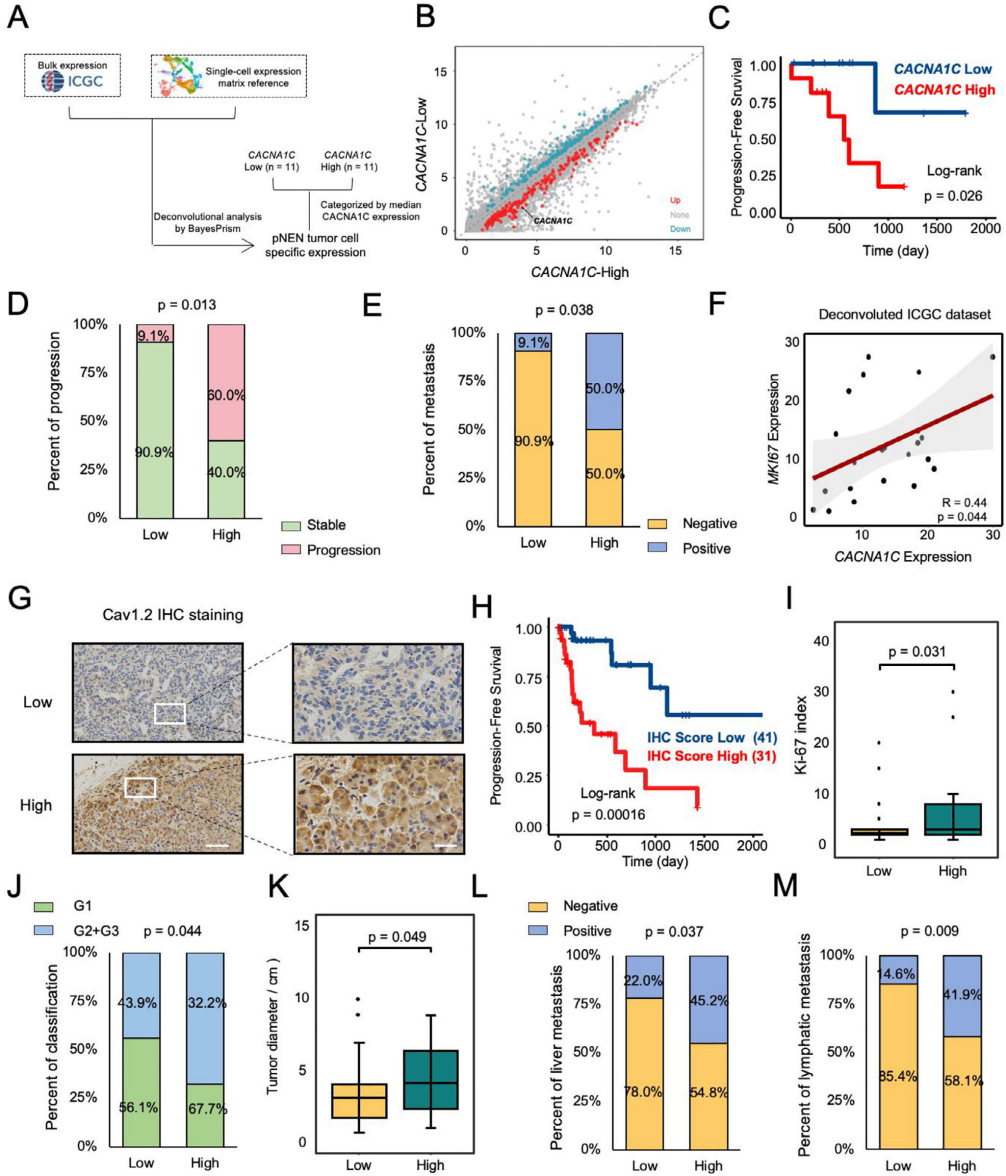
Cav1.2 correlates with the malignant biological behaviors of pNEN. (A) Schematic depicting deconvolutional analysis of the ICGC dataset for tumor cell‐specific expression matrix and categorized as *CACNA1C* low and *CACNA1C* high groups based on median expression of *CACNA1C*. (B) Dot plot of DEGs between the *CACNA1C* low and the *CACNA1C* high group. A logarithmic expression was presented. (C) Kaplan‐Meler analysis of the PFS rate between the *CACNA1C* low and high groups. *P*‐value was calculated by the Log‐rank test. (D, E) Proportional analysis of disease progression state (D) and distal metastasis rate (E) between two groups. *P*‐value was calculated by the chi‐square test. (F) Positive correlations between tumor cell‐specific expression of *CACNA1C* and *MKI67* in the deconvoluted ICGC dataset. *P*‐value was calculated by spearman correlation test. (G) Representative images of Cav1.2 IHC staining of low (top) and high (bottom). Scale bars: 50 µm for lower magnification and 10 µm for higher magnification. (H) Kaplan‐Meler analysis of PFS rate according to the Cav1.2 IHC score. *P*‐value was calculated by the Log‐rank test. (I) Clinically evaluated the Ki‐67 index among the Cav1.2 IHC score high or low group. ^*^ *p* < 0.05 according to unpaired two‐tailed Student's t‐test. (J) Proportion of tumor grade of pNEN evaluated in each group. *P*‐value was calculated by the chi‐square test. (K) Boxplot of tumor diameter in the Cav1.2 IHC score high or low group. The longest diameter of the tumor was considered for analysis. ^*^ *p* < 0.05 according to unpaired two‐tailed Student's t‐test. The box represents the interquartile range; whiskers are 1.5× the interquartile range. (L, M) Proportional analysis of liver metastasis rate (L) and lymphatic metastasis rate (M) between two groups. *P*‐value was calculated by the chi‐square test.

Next, we analyzed correlations of *CACNA1C* expression with clinical outcomes to further excavate the potential role in promoting disease progression. First, we performed deconvolutional analysis of a bulk RNA‐seq dataset from the ICGC database (https://dcc.icgc.org/releases/current/Projects). By mapping the tumor cell annotations with scRNA‐seq reference, we obtained a tumor cell‐specific expression matrix corresponding to the ICGC dataset and then categorized it into two groups based on the median *CACNA1C* expression (Figure 4A). DEG analysis (223 up‐regulated genes and 217 down‐regulated genes) revealed the distinctive features among the two groups (Figure 4B; Figure S4A).

For deconvoluted ICGC datasets, the results of survival analysis showed that the patients with higher tumor cell‐specific *CACNA1C* expression were observed to have less PFS (Figure 4C). Besides, we observed that, compared with the lower expression group (*n* = 11), the higher expression group (*n* = 10 due to one patient with missing information) was correlated with a higher percentage of disease progression (Figure 4D, 60.0% vs 9.1%) and distal metastasis (Figure 4E, 50.0% vs 9.1%). We also found that tumor cell‐specific *CACNA1C* expression positively correlated with *MKI67* expression (Spearman R = 0.44, p = 0.044), suggesting that high *CACNA1C* expression links with enhanced tumor proliferation potential (Figure 4F). These data indicate that Cav1.2 correlates with poor clinical outcomes.

To make further validation, we evaluated the tumor cell‐specific *CACNA1C* expression in our cohort (*n* = 72) by IHC and analyzed the relationship with clinical outcomes. Consistently, we found the same correlation between tumor cell‐specific *CACNA1C* expression and PFS (Figure 4G,H). Patients with higher expression of *CACNA1C* correlated with higher Ki‐67 index (Figure 4I) and higher percentage of high (G2 or G3) grade (Figure 4J), respectively. Tumor diameter also significantly differed (Figure 4K). Moreover, patients with higher expression of *CACNA1C* correlated with a higher percentage of liver progression (Figure 4L, 45.2% vs 22.0%) and lymphatic metastasis (Figure 4M, 41.9% vs 14.6%). Combining these results, we concluded that Cav1.2 may be a strong indicator for pNEN with malignant biological behaviors.

### 2.5 Cav1.2 Induces Local H3K27 Acetylation by Recruiting P300/CBP via CaM‐CAMKII‐CREB1 Pathway

Since we found the epigenetic control of H3K27ac for *CACNA1C*, it was then shown at the *CACNA1C* gene locus that there were more apparent peaks in the pNEN group rather than the normal group (Figure S5A). Moreover, ChIP‐qPCR revealed the local occupancy of H3K27ac, CBP, and P300 (Figure S5B). Treatment of A485 or C646 significantly diminished Cav1.2 level (Figure S5C). These results reinforce the regulatory targets of H3K27ac and its key modification enzyme, P300/CBP, on *CACNA1C*.

**FIGURE 5:**
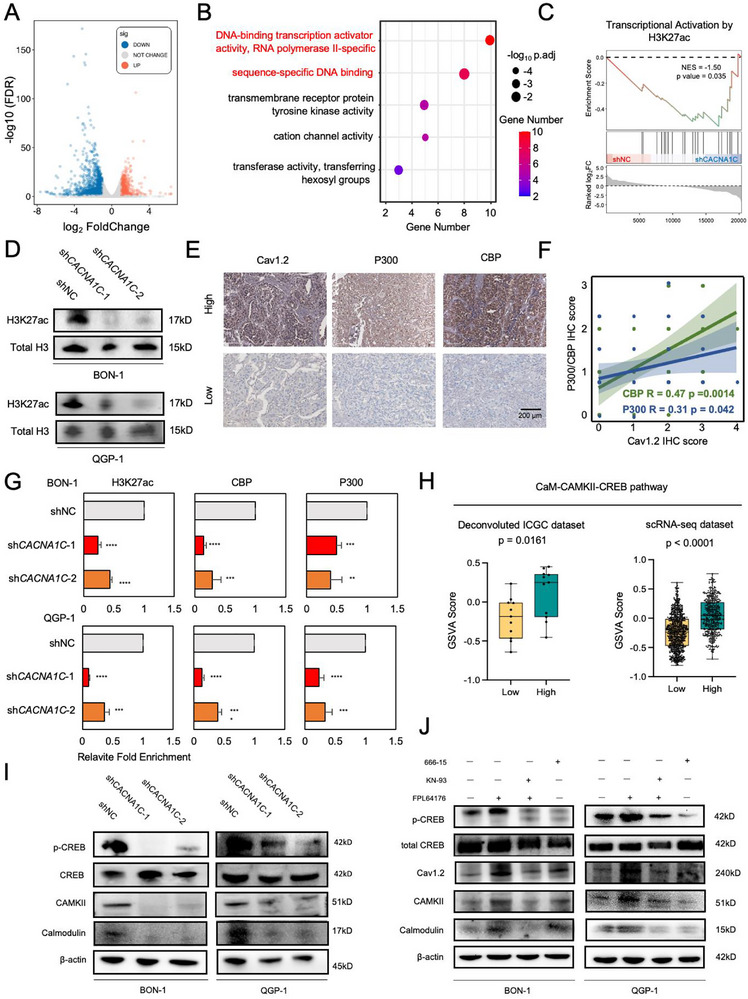
Cav1.2 forms a positive regulatory circuit with H3K27ac via the CaM‐CAMKII‐CREB1 pathway. (A) Volcano plot showing DEGs between the control and stable knockdown group in QGP‐1 cells. (B) GO analysis showing the most enriched molecular function terms (red) in down‐regulated DEGs. (C) GSEA analysis of transcriptional activation by H3K27ac geneset in control or *CACNA1C* stably knockdown QGP‐1 cells. (D) Western Blots of global H3K27ac among the control and two independent knockdown groups. Total H3 was treated as a reference. (E) Representative images of CBP/P300 and Cav1.2 IHC staining in low and high groups according to Cav1.2 IHC score. Scale bar: 200 µm. (F) Quantification of IHC scores revealed the positive correlations between P300/CBP *with* Cav1.2. *P*‐value was calculated by spearman correlation test. (G) ChIP‐qPCR showing relative enrichment of H3K27ac, P300/CBP at *CANCA1C* locus. ^**^ *p* < 0.01; ^***^ *p* < 0.001; ^****^ *p* < 0.0001 according to one‐way ANOVA with Tukey's multiple comparisons tests. Data were shown as mean ± SD. Data were shown as mean ± SD. (H) Boxplot of GSVA score corresponding to CaM‐CAMKII‐CREB1 pathway calculated in both deconvoluted ICGC dataset (left) and scRNA‐seq dataset (right). The box represents the interquartile range; whiskers are 1.5× the interquartile range. (I) Western blots showing alterations in CaM, CAMKII, and phosphorylated CREB1 related to the target pathway. (J) Western blots showing alterations in CaM, CAMKII, Cav1.2, and phosphorylated CREB1 under different treatments.

Next, we aimed at the downstream of *CACNA1C*. We performed transcriptomic sequencing on stably knockdown of *CACNA1C* and normal control in QGP‐1 cells. There were 2284 differentially expressed genes (including 844 up and 1440 down‐regulated genes) in total (Figure 5A). GSEA analysis also showed significantly down‐regulated genes on the calcium signaling pathway (Figure S5D), suggesting that knockdown successfully silenced intracellular calcium signaling. Down‐regulated genes were selected and subjected to GO analysis, which showed that impaired transcriptional factors binding activity (Figure 5B). Further analysis of the gene set of transcriptional activation by H3K27ac showed negative enrichment in stably knockdown of *CACNA1C* cells (Figure 5C). Considering the consequence of transcriptional factors binding that induced the active chromatin architecture, which was marked by H3K27ac, we legitimately hypothesized whether *CACNA1C* knockdown altered the global H3K27ac level. As expected, western blots revealed the dramatic decrease of global H3K27ac comparing the knockdown with the control group (Figure 5D). However, the key enzyme, P300/CBP, seemed to be stable after *CACNA1C* knockdown (Figure S5E,F), indicating the impaired recruitment of P300/CBP after *CACNA1C* silencing. Simultaneously, we detected the expression level of *CACNA1C* and key modification enzymes, P300/CBP, from pNEN samples in our cohort by IHC. A positive expression correlation of P300/CBP with Cav1.2 was observed (Figure 5F), suggesting potential regulatory partnerships for *CACNA1C* with epigenetic modulation. We then found that after *CACNA1C* knockdown, the occupancy of H3K27ac, CBP and P300 at *CACNA1C* gene locus significantly decreased (Figure 5G), indicating the local dislodgement of H3K27ac and P300/CBP. Therefore, we concluded that Cav1.2 regulates local epigenetic modification of H3K27ac.

It was widely reported that Cav1.2 functioned via the CaM‐CAMKII‐CREB1 pathway, which finally led to CREB1 phosphorylation and enhanced P300/CBP binding [31]. We then considered whether Cav1.2 regulated local epigenetic modification of H3K27ac via this pattern. We first calculated Gene Set Viriation Analysis (GSVA) score both in deconvoluted ICGC dataset and scRNA‐seq dataset, and then found that the higher of *CACNA1C* expression, the more activated and enriched in this pathway (Figure 5H). Then we calculated the interaction score of CREB1 according to the STRING (https://cn.string‐db.org) database; the top two interacting proteins were CBP and P300 (Figure S5G). Co‐IP assay revealed that the interaction of CREB1 with P300/CBP was lost compared with the control group (Figure S5H,I). We next validated the protein level of corresponding molecules. It was found that after *CACNA1C* knockdown, the protein level of CaM (Calmodulin) and CAMKII decreased significantly, as well as the phosphorylated CREB1 (Figure 5I). Additionally, we introduced several small molecular modulators, FPL64176 as a Cav1.2 agonist [32], KN‐93 as a CAMKII inhibitor, and 666‐15 as a CREB1 inhibitor. By adding FPL64176 alone, we found an increased level of CaM, CAMKII, Cav1.2 itself, as well as phosphorylated CREB1. When combining with either KN‐93 or 666‐15, we observed a decreased level of CaM, CAMKII, Cav1.2, and phosphorylated CREB1, compared with adding FPL64176 alone (Figure 5J). Taken together, we proposed that Cav1.2 induces local H3K27ac modification at the *CACNA1C* gene locus by recruiting P300/CBP via the CaM‐CAMKII‐CREB1 pathway, thus forming a positive regulatory circuit.

### 2.6 Calcium Channel Blockers Inhibit pNEN Progression

**FIGURE 6:**
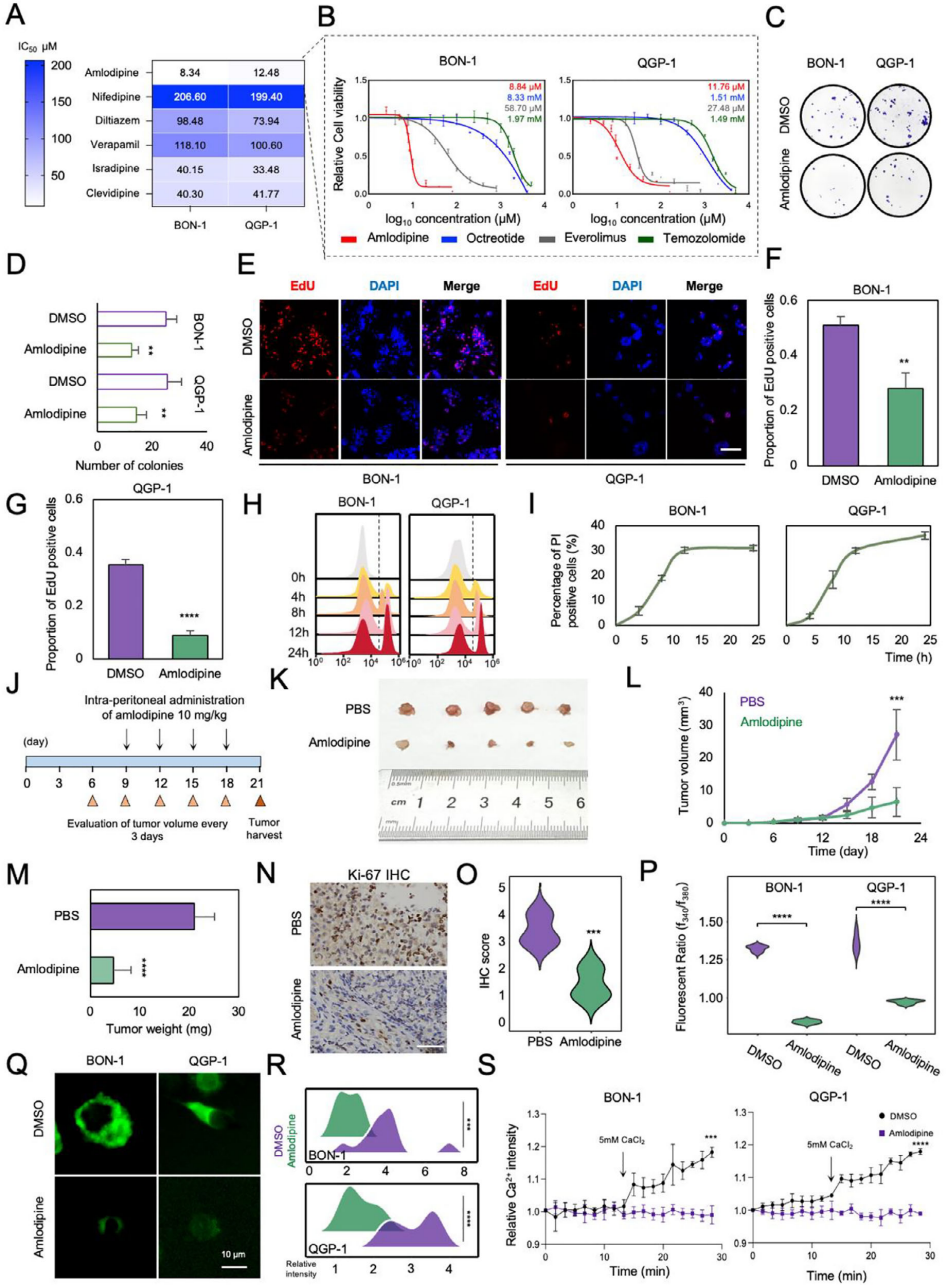
Calcium channel blockers inhibit pNEN progression. (A) Heatmap showing IC_50_ among different CCBs in BON‐1 and QGP‐1 cells. (B) Comparison of IC_50_ between amlodipine, octreotide, everolimus and temozolomide in BON‐1 and QGP‐1 cells. (C, D) Representative images showing colonies of the control and amlodipine treatment group (C) and quantification statistics (D). ^**^ *p* < 0.01; ^***^ *p* < 0.001; ^****^ *p* < 0.0001 according to unpaired two‐tailed Student's t‐test. Data were shown as mean ± SD. (E–G) Representative images of EdU staining (E) and quantification of EdU‐positive cells per field in BON‐1 (F) and QGP‐1 (G) cells. Scale bar: 100 µm. ^**^ *p* < 0.01; ^***^ *p* < 0.001; ^****^ *p* < 0.0001 according to unpaired two‐tailed Student's t‐test. Data were shown as mean ± SD. (H, I) Histogram of flow cytometry of propidium iodide (PI) in different time series of amlodipine treatment in BON‐1 and QGP‐1 cells (H) and quantification of percentage of dead cells (I). (J) Schematic showing the strategy of amlodipine administration in the BON‐1 CDX model. Intraperitoneal administration of amlodipine began at nine days after inoculation; daily dosage was 10 mg/kg. (K) Macroscopic presentation of tumors in the PBS and amlodipine treatment group. (L) Tumor volume was calculated every three days post inoculation of BON‐1 cells. ^***^ *p* < 0.001 according to unpaired two‐tailed Student's t‐test. Data were shown as mean ± SD. Sample size *n* = 5. (M) Tumor weight was measured in the PBS and amlodipine treatment groups. ^****^ *p* < 0.0001 according to unpaired two‐tailed Student's t‐test. Data were shown as mean ± SD. Sample size *n* = 5. (N) Representative images of IHC staining of the Ki‐67 for CDXs among the control and knockdown groups. Scale bar: 20 µm. Sample size *n* = 5 for each group. (O) Statistics regarding Ki‐67 IHC staining. ^***^ *p* < 0.001 according to unpaired two‐tailed Student's t‐test. Sample size *n* = 5. (P) Intracellular calcium concentration measured in BON‐1 and QGP‐1 cells. Amlodipine significantly lowered the intracellular calcium level. ^****^ *p* < 0.0001 according to unpaired two‐tailed Student's t‐test. Sample size *n* = 5. (Q) Calcium imaging of GCaMP6s in BON‐1 and QGP‐1 cells. Amlodipine lowered the cytosolic calcium level. Scale bar: 10 µm. Sample size *n* = 3. (R) Quantification of cytosolic calcium decrease on amlodipine treatment. ^***^ *p* < 0.0001 according to unpaired two‐tailed Student's t‐test. (S) Extracellular calcium flux assay. 5 mm CaCl_2_ was added as extracellular calcium. Influx of calcium was partially blocked after amlodipine treatment. ^***^ *p* < 0.001; ^****^ *p* < 0.0001 according to unpaired two‐tailed Student's t‐test. Data were shown as mean ± SD.

Since Cav1.2 was identified as the crucial target responsible for pNEN progression and progressive behaviors with tumor‐specific expression, we next reflected on the possible way to retard pNEN progression by blocking this target. CCBs were a series of small molecules that were capable of blocking calcium channels, which were widely prescribed as anti‐hypertensive drugs and anti‐arrhythmic drugs with outstanding efficacy and safety [21]. We were inspired by these findings to explore whether CCBs could be used against pNEN progression by blocking Cav1.2. First, we selected several commonly used CCBs to test their half‐maximal inhibitory concentration (IC_50_) in BON‐1 and QGP‐1. Among six types of CCBs, we found that amlodipine was the most effective candidate for inhibiting the growth of pNEN cells (Figure 6A). Next, we took octreotide, the first‐line anti‐proliferative agent for G1 and G2 pNETs, and the other two commonly used drugs for pNEN, everolimus and temozolomide, as controls. It was shown that, in both BON‐1 and QGP‐1, IC_50_ of the amlodipine was significantly lower than that of octreotide, everolimus, and temozolomide (Figure 6B), suggesting amlodipine may exert better therapeutic efficacy compared with existing drugs. Meanwhile, we also included pancreatic adenocarcinoma (PDAC), BxPC‐3, SW1990, CFPAC‐1, and normal pancreatic ductal cell HPDE6‐C7 to test their IC_50_ to amlodipine. Results showed that their IC_50_ to amlodipine was about 40 µm (Figure S6A), much higher than that in BON‐1 or QGP‐1. This indicated a specific mechanism on pNEN cell of calcium signaling, not in PDAC.

Next, we focused on amlodipine and explored its inhibitory effect on pNEN. For the in vitro assay, we observed a reduced number of clones in the amlodipine treatment compared with the control group (DMSO) in the colony formation assay (Figure 6C,D). EdU assay also showed a decrease of positive cells (Figure 6E–G), indicating that amlodipine potently blocked cellular proliferation. On the other aspect, we also analyzed the cytotoxic effect of amlodipine. By using propidium iodide (PI) staining followed by flow cytometry to evaluate dead cells, we found that amlodipine induced pNEN cell death at time‐dependent manner, with about 12 h to reach the maximum cytotoxic effect (Figure 6H,I). Another way we selected to assess the cytotoxicity was the lactate dehydrogenase (LDH) release assay. Similarly, the percentage of dead cells (assessed by LDH release), increased in a time‐dependent manner (Figure S6B). Meanwhile, we also tested the specificity of amlodipine for targeting Cav1.2. After amlodipine treatment, both of the two Cav1.2 agonists Bay k8644 [33] and FPL64176 neutralized the anti‐proliferative effect of amlodipine (Figure S6C). When treating cells with these two agonists independently, a similar effect for promoting cell proliferation was observed (Figure S6D). These data indicate that amlodipine may exert an inhibitory effect in pNEN by specifically blocking Cav1.2, not other calcium channels.

For the in vivo assay, through the CDX model, amlodipine was intraperitoneally administrated to mice 9 days after subcutaneous seeding of BON‐1 cells (Figure 6J). We found that the tumor size was significantly smaller in the amlodipine group compared with PBS (Figure 6K). The tumor volume and tumor weight were also decreased in the amlodipine comparing with the PBS group (Figure 6L,M), while the body weight among the two groups was not different (Figure S6E). Moreover, IHC staining of Ki‐67 for CDXs also showed the retarded proliferation rate in the amlodipine treatment group (Figure 6N,O). These data suggest that amlodipine may be one of the most effective CCBs targeting Cav1.2, which inhibit pNEN progression in vitro and in vivo.

Next, to clarify the detailed mechanism of amlodipine functions, we first tested the molecular alterations described above. We found that H3K27ac and the CaM‐CAMKII‐CREB1 pathway were lowered in protein level (Figure S6F), both in BON‐1 and QGP‐1. Additionally, it was further validated that amlodipine reduced intracellular calcium concentration (Figure 6P). Calcium imaging by GCaMP6s also showed a decrease in fluorescent intensity of cytosolic calcium after amlodipine treatment (Figure 6Q,R). Moreover, amlodipine significantly blocked calcium influx in BON‐1 and QGP‐1 cells (Figure 6S). Those alterations after amlodipine treatment were consistent with the mechanism of a positive regulatory circuit between Cav1.2 and H3K27ac. Taken together, these results indicate that amlodipine exerts an inhibitory effect on pNEN and may be superior to octreotide. Mechanistically, amlodipine disrupts the positive regulatory circuit between Cav1.2 and H3K27ac, all of which may propose a new therapeutic option for inhibiting pNEN progression.

### 2.7 Clinical Administration of Calcium Channel Blockers Correlates With Improved Progression

**FIGURE 7:**
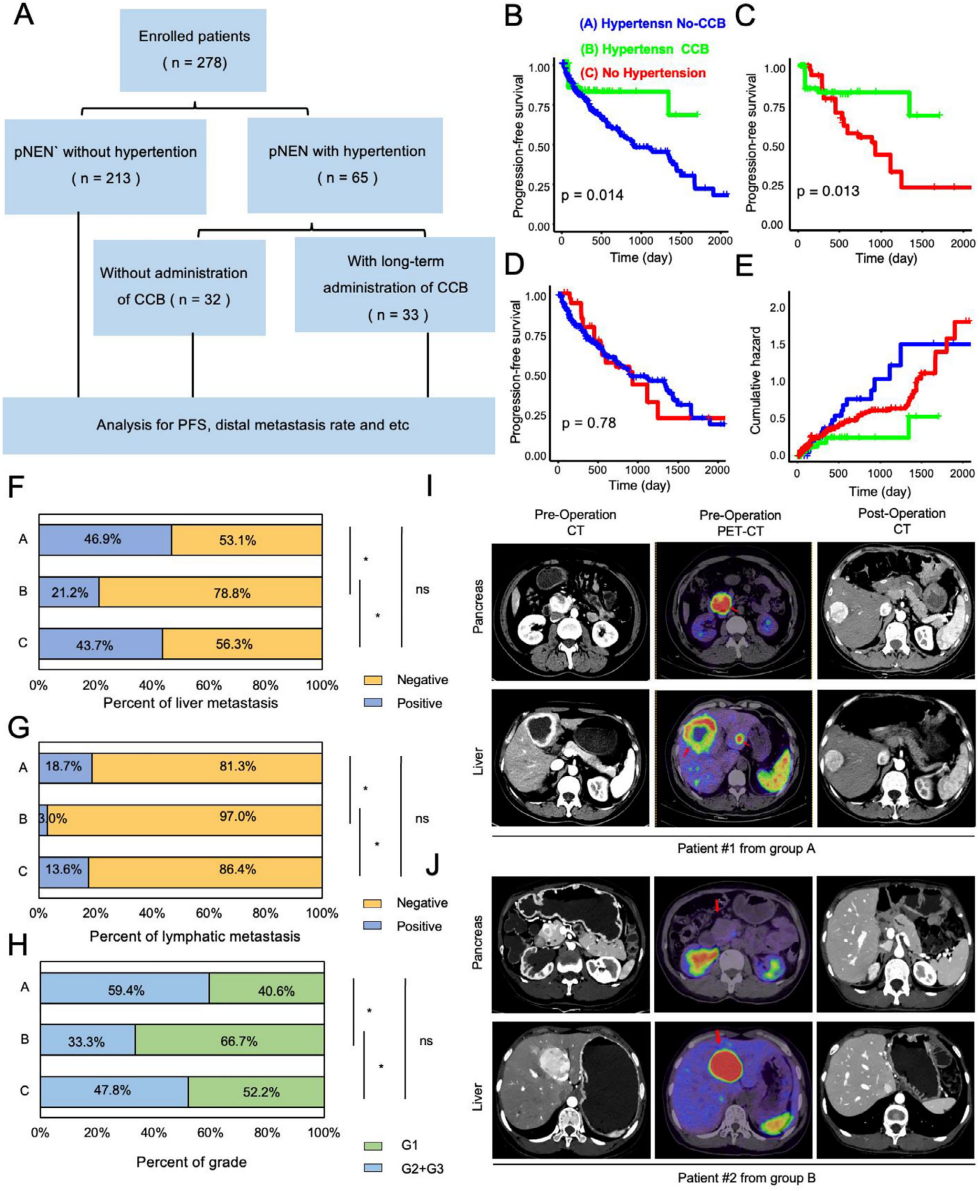
Clinical administration of calcium channel blockers correlates with improved progression. (A) Flowchart delineating clinical data analytic process. (B–D) Kaplan‐Meler analysis of PFS rate between the hypertension without CCB and hypertension with CCB group (B), between hypertension with CCB and no hypertension group (C), as well as between hypertension without CCB and no hypertension group (D). (E) Cumulative risk curves among hypertension without CCB (blue), hypertension with CCB (green), and no hypertension group (red). (F, G) Proportional analysis of liver metastasis rate (F) and lymphatic metastasis rate (G) among the three groups mentioned above. *P*‐value was calculated by the chi‐square test. (H) Proportional analysis of disease grading among the three groups mentioned above. *P*‐value was calculated by the chi‐square test. (I, J) Representative images of CT and ^68^Ga PET‐CT before or after operation in two patients corresponding to group A (I, hypertension without CCB administration) and group B (J, hypertension with CCB administration).

To further explore the inhibitory role of CCBs on pNEN, we enrolled the pNEN cohort, which contained patients with or without hypertension; the former could be sub‐classified as patients with or without CCBs administration. Those patients were evaluated with PFS and distal metastasis rate (Figure 7A). We first balanced the baseline characteristics among three groups with Inverse Probability of Treatment Weighting (IPTW). After IPTW balancing, the age, BMI, and AJCC stage variables were adjusted, and all the variables in the baseline were balanced and comparable (Figure S7A). IPTW‐corrected survival analysis indicated that PFS was significantly higher in hypertension with the CCB group compared to both the hypertension without CCB group and the no‐hypertension group (Figure 7B,C), and there was no significant difference between the hypertension without CCB group and the no‐hypertension group (Figure 7D). Meanwhile, the cumulative hazard of hypertension without the CCB group and no‐hypertension group was significantly higher than that of hypertension with the CCB group (Figure 7E; Figure S7B,C), indicating the lower risk of disease progression and the potential benefit of CCB administration.

Moreover, the liver metastasis rate in hypertension with the CCB group was significantly lower than the other two groups (Figure 7F), and a similar result was observed for the lymphatic metastasis rate (Figure 7G). In addition, the percentage of high grade (G2+G3) was significantly lower than that of the two groups (Figure 7H), indicating that administration of CCB correlated with a lower level of tumor proliferation state. 2 patients, representing group A and group B was shown with pre‐operative CT/^68^Ga PET‐CT or post‐operative CT images. Patient #1 from group A was considered as disease progression since the post‐operative relapse in the liver was observed (Figure 7I), while patient #2 from group B was considered as progression‐free (Figure 7J).

Collectively, we conclude that clinical administration of CCB correlates with better PFS and lower liver or lymphatic metastasis rate, indicating the potential therapeutic efficacy of CCB administration to pNEN patients.

## 3 Discussion

pNEN is highly heterogeneous, which is reflected in the variable clinical manifestations and differential grading. Disease progression of pNEN is commonly seen in clinical practice, raising the problems of difficulty and complexity of treatment, as well as shortened overall survival (OS) [2]. Several mechanisms have been demonstrated that contribute to pNEN progression, such as aberrant PI3K‐Akt‐mTOR activation and microvascular genesis. Advanced therapeutic agents targeting these two pathways, everolimus and sunitinib (multi‐targeted receptor tyrosine kinase inhibitor) was approved to treat low‐grade pNEN and were capable of improving progression‐free survival [34, 35, 36]. However, a 5‐year OS benefit was not observed, and significant adverse effects were reported, making it difficult for disease in the long term. Other mechanisms, such as *MEN1* mutation, *DAXX/ATRX* dysfunction, and associated ALT, were only identified as risk factors associated with poor prognosis or disease progression, but clinical evaluations were still not well‐established, and there were no relevant therapeutic agents targeting these processes so far. Argent needs of new mechanisms and corresponding therapeutic targets are required for investigation since the incidence as well as the emphasis toward pNEN rises.

Epigenetic modifications have been extensively investigated in cancers, as the key role in carcinogenesis, malignant transformation, and tumor immune evasion [37, 38]. Development of therapeutic approaches targeting cancer epigenetics is one of the most popular focuses. One recent case from histone H3 lysine trimethylation (H3K27me3) was screened and selected as the epigenetic drivers in adult T cell leukaemia/lymphoma (ATL) [39]. Two phases of clinical trial, evaluating the efficacy of Valemetostat, a potent, novel, dual inhibitor of EZH2 and EZH1, have shown satisfying results that lead to both durable responses for patients with relapsed or refractory ATL and a manageable safety profile [40, 41]. However, it is still worth noting that dose‐limiting toxicity and lack of selectivity, both in tissue level and gene level, are the most prominent issues limiting the application in the clinical arena [42]. Another issue that raises concerns is that the most pre‐clinical and clinical researches focus on the hematopoietic cancers as they are clearly susceptible to epigenetic interventions [42]. These issues still lack a detailed investigation.

Calcium channels serve as one of the most pivotal roles in neurotransmission and cardiac conduction. Cav1.2 aberrations are previously reported to be pathogenic in schizophrenia and bipolar disorder, as well as Brugada syndrome, which is considered a type of cardiac arrhythmias, with possible mechanisms of abnormal Cav1.2 leading to excessive influx of calcium, and corresponding pathological alterations. Recent studies have demonstrated that Cav1.2 is expressed and functions in tumorigenesis or cellular chemosensitivity, indicating the significance of calcium homeostasis in cancer development and therapeutics [20]. A recent study has mentioned a type of calcium channel, *CACNA1D*, in NF‐pNETs, which serves as a subtype‐specific marker and is sensitive to amiodarone treatment [43]. However, there are no reports so far clarifying the in‐depth significance of calcium signaling as well as relevant molecules in pNEN. We are the first to uncover the crucial role of calcium signaling and calcium channel Cav1.2 in promoting pNEN progression and being a strong indicator of progressive behaviors in pNEN, which broadens our horizon of new therapeutic targets in pNEN.

Concerning the efficacy and safety of CCBs against cardiovascular diseases, it has been widely administered for decades among patients. Recent studies have also demonstrated the unconventional indications. Regarding the calcium channel blockade properties, it is tested against mental diseases and has shown satisfying outcomes [44]. Moreover, it is also reported that CCBs are capable of regulating chemosensitivity. For instance, it is shown that Cav1.2‐positive mesenchymal stem cells existing in the bone marrow of patients with acute myeloid leukemia (AML) are the key to disease progression, and lercanidipine can disrupt leukemia progression if used in combination with chemotherapy [45]. In addition, amlodipine is reported to enhance gemcitabine sensitivity in PDAC [46]. Hence, these evidences indicate the potential role of CCBs against other types of diseases, especially cancer, which is characterized by abnormal calcium channels. In the same way, regarding pNEN, we have elaborated on the efficacy of CCBs (especially amlodipine) against pNEN progression, and the results strongly indicate that administration of CCBs can be considered as an attractive therapeutic option against pNEN progression.

There are still potential limitations; however, amlodipine was identified as the most effective CCBs against pNEN in our study, the equivalent dosage of which for humans is much higher than the commonly used dosage in clinical practice. The lowest dosage of amlodipine with anti‐proliferative efficacy against pNEN may need further investigation. Since we observed correlations between CCBs administration and improved PFS as well as distal metastatic rate, it was not included that inhibition of other types of calcium channels may contribute as well due to relatively non‐specific Cav1.2 inhibition. Clinical investigations and molecular mechanisms among different calcium channels or their downstream regulatory networks in pNEN may also warrant further study.

Overall, the current findings emphasize the novel mechanism of the positive regulatory circuit of calcium signaling with epigenetic control by H3K27ac. Cav1.2, the key target in calcium signaling, is identified to be specifically expressed in pNEN cells and contributes to pNEN progression. Mechanistically, we demonstrate that Cav1.2 regulates local H3K27 acetylation by recruiting P300/CBP via the CaM‐CAMKII‐CREB1 pathway, which forms a positive regulatory circuit with H3K27ac. Further analyses highlight that CCBs strongly inhibit pNEN progression in both experimental and clinical aspects. As a result, this study provides a new insight into therapeutic targets and the potential approach against pNEN progression.

## 4 Methods

### 4.1 Human Participants

Clinical data of 278 pNEN patients were collected and analyzed. Among them, a total of 44 paired paraffin‐embedded adjacent normal and pNEN tissues were collected for tissue microarray preparation. 28 additional pNEN tissues were also collected. All samples were immediately fixed with paraformaldehyde after surgery. Evaluation of disease state was based on the Response Evaluation Criteria in Solid Tumors (RECIST1.1).

### 4.2 Cell Lines

BON‐1 was cultured in DMEM/F12 supplemented with 10% FBS and 1% penicillin/streptomycin. QGP‐1, BxPC‐3, SW1990, and CFPAC‐1 were cultured in RPMI‐1640 supplemented with 10% FBS and 1% penicillin/streptomycin. 293T was cultured in DMEM supplemented with 10% FBS and 1% penicillin/streptomycin. Cells were cultured at 37°C under 5% CO_2_ in the air.

### 4.3 Animals and Animal Experiments

BALB/c nude mice (6–8 weeks old) were purchased from the animal husbandry center of Sun Yat‐Sen University. Mice were randomized and maintained under specific‐pathogen‐free conditions with proper circumstances in the animal facility with free access to water and food.

For BON‐1 cell transplantation, 1 × 10^7^ cells was harvested and resuspended with 50% vol of Matrigel in PBS. 100 µL of suspension was injected into the axillary fossa of female nude mice.

For amlodipine treatment, each mouse started to receive intraperitoneal injections at a concentration of 10 mg/kg [47, 48] every three days, nine days after the inoculation of tumor cells. Doses of PBS or amlodipine injections were continued throughout the experiment.

Tumor volumes were measured using a vernier caliper and calculated as (length × width^2^)/2. At the experiment endpoint (21 days after inoculation), the cell proliferation was validated by IHC.

### 4.4 Protein Extraction and Western Blots

Cells were harvested and lysed in RIPA lysis buffer at 4°C for 30 min. Lysate was separated on SDS‐PAGE gel and then wet transferred to a 0.45 µm PVDF membrane in ice‐cold transfer buffer for 3 h. After blocking with 5% non‐fat milk in TBST for 1 h at room temperature, the membrane was incubated with the primary antibody overnight at 4°C. After washing three times with TBST, the membrane was incubated with 1:5000 secondary antibodies for 1 h at room temperature. After another wash for three times with TBST, the membrane was incubated with supra‐ECL substrate and imaged using a CCD camera.

### 4.5 RNA Isolation and qRT‐PCR

Total RNA was extracted from the specified cells using Trizol according to the manufacturer's instructions. Reverse transcription and RT‐qPCR were performed by HiScript II Q RT SuperMix for qPCR and ChamQ Universal SYBR qPCR Master Mix, respectively. QuantStudio 6 Flex Real‐Time PCR Systems (Applied Biosystems) were used to quantify relative mRNA expression levels, and the data were analyzed using the 2^−ΔΔCT^ algorithm. Primers used are listed in Table S3.

### 4.6 Intracellular Calcium Measurement

6 × 10^4^ cells were seeded in 96‐well plates for each group. Cells were washed with Hank's Balanced Salt Solution (HBSS) three times. 100 µL of calcium detection buffer (2 µm Fura‐2AM and 0.05% Pluronic F‐127 in HBSS) was added per well, and cells were incubated for 60 min at 37°C. After incubation, cells were washed again with HBSS three times. Another 100 µL of HBSS was added per well, and 5 mm CaCl_2_ was added if necessary. Intracellular calcium concentration was measured consecutively by Microplate Reader at excitation wavelength of 340 and 380 nm, and emission wavelength of 510 nm.

### 4.7 GCaMP6s for Calcium Imaging

To measure intracellular calcium intensity in BON‐1 and QGP‐1 cells, the calcium indicator GCaMP6s was transiently expressed in target cells. Imaging was conducted 24 h after transfection, and green fluorescence intensity (488 nm laser sources) was measured by NIS‐Elements imaging software of Nikon Fluorescent microscope.

### 4.8 IHC

IHC was performed as described previously [49]. IHC scores were recorded by two experienced pathologists independently as staining intensity (negative, 0; weak, 1; moderate, 2; strong, 3) multiplying stain area (negative, 0; ≤30%, 1; >30 and ≤60%, 2; >60%, 3) [50].

### 4.9 IF

Slides were stained as previously described [51]. Secondary antibody was diluted 1:200. After secondary antibody incubation for 1 h, slides were washed three times and then stained with 1 µg/mL DAPI in PBS for 10 min at room temperature without light exposure. Finally, images were acquired at the Zeiss Confocal microscope LSM 800. Images were post‐processed using Fiji Is Just ImageJ (FIJI) 2.9.0.

### 4.10 Single Molecular TIRF Imaging

Cells were previously seeded into a confocal dish and incubated with 75 mm CaCl_2_ or H_2_O for 24 h. Cells were then washed with PBS and fixed with 4% PFA for 15 min at room temperature. Following 3× washes in PBS, cells were incubated for 10 min using 0.5% Triton‐X‐100, followed by blocking with 5% BSA. After removal of the blocking solution, cells were incubated with 1:200 anti‐Cav1.2 overnight at 4°C. Cells were then washed 3× for 5 min with PBS before being incubated with a secondary antibody, anti‐rabbit IgG 488 (1:400). After 3× washes for 5 min in PBS, cells were stained with DAPI for 15 min at room temperature and stored at 4°C until imaging was performed.

Images were obtained using the Leica microscope coupled to a TIRF module for single‐molecular imaging. Images were acquired using a 488 nm excitation laser and appropriate emission filters with default parameters. Images were post‐processed using FIJI 2.9.0.

### 4.11 ChIP

1 × 10^6^ cells were harvested and fixed with formaldehyde at 1% final concentration for 15 min at room temperature. Glycine was added to terminate fixation. Fixed cells were then resuspended and lysed with lysis buffer (10 mm Tris HCl, pH 7.5, 50 mm NaCl, 5 mm MgCl_2_, 5 mm CaCl_2_, 0.2% NP‐40, and 1× Proteinase Inhibitor Cocktail) for 10 min at 4°C. Lysis was then fragmented by MNase for 20 min at 37°C. MNase was inactivated by adding 0.8 µL of 0.5 m EGTA with incubation at 65°C for 10 min. Reverse‐crosslinking buffer (1% SDS, 0.01 mg/mL RNase A, 200 mm NaCl, and 1 mg/mL Proteinase K in 1×TE) was then added and supernatant was collected. Fragments were then incubated with 5 µg of the indicated antibody pre‐bound to Protein A/G magnetic beads. After incubation, beads were washed twice with 1× TE buffer. Fragments were then extracted by equal volume of PCI: A and collected by Universal DNA Purification Kit under the manufacturer's instructions. qRT‐PCR was performed similarly to what was described above. The normal IgG‐tagged group was assigned as the negative control.

### 4.12 Co‐IP

Nuclear extracts were prepared for control and two independent *CACNA1C* knockdown cells. Nuclear extracts were immunoprecipitated with 5 µg of Anti‐CREB1 antibody pre‐bound to Protein A/G magnetic beads and immunoblotted with P300 and CBP.

### 4.13 Colony Formation Assay

1 × 10^3^ cells were sparsely seeded in 6‐well plates for each group, cultured for one week, fixed with paraformaldehyde, and stained with 0.1% crystal violet in methanol for 10 min at room temperature. The colony number was counted manually.

### 4.14 CCK8 Cell Proliferation

6 × 10^4^ cells were seeded in 96‐well plates for each group, 10 µL of Cell Counting Kit‐8 was added to each well, and incubation at 37°C for 1 h. Cell viability was measured with a Microplate Reader at 450 nm absorbance.

### 4.15 RNA‐Seq

Total RNA was extracted from the samples using Trizol. The RNA quality was checked by Agilent 2200, where RIN (RNA integrity number) > 7.0 is acceptable for cDNA library construction. The cDNA libraries were constructed for each RNA sample using the VAHTS Universal V6 RNA‐seq Library Prep Kit for Illumina (Vazyme) according to the manufacturer's instructions. Briefly, the protocol consists of the following steps: Poly‐A containing mRNA was purified from 1µg total RNA using oligo(dT) magnetic beads and fragmented into 200–600 bp using divalent cations at 85°C for 6 min. The cleaved RNA fragments were used for first‐ and second‐strand complementary DNA (cDNA) synthesis. dUTP mix was used for second‐strand cDNA synthesis, which allows for the removal of the second strand. The cDNA fragments were end‐repaired, A‐tailed, and ligated with indexed adapters. The ligated cDNA products were purified and treated with uracil DNA glycosylase to remove the second‐strand cDNA. Purified first‐strand cDNA was enriched by PCR to create the cDNA libraries. The libraries were quality controlled with Agilent 2200 and sequenced by DNBSEQ‐T7 on a 150 bp paired‐end run.

### 4.16 Propidium Iodide Staining and Flow Cytometry

The death cell analysis by propidium iodide (PI) staining was determined according to the previous studies [52]. Cells previously seeded into a 6‐well plate were harvested and washed twice with PBS, then cells were stained with PI (10 µg/mL, TargetMol) for 15 min at RT, avoiding light. No treatment was set as control. After staining, the proportion of death cell was analyzed by the CytoFLEX system (Beckman). Data was analyzed by CytoExpert 2.4.

### 4.17 LDH Cytotoxicity Assay

The cytotoxicity analysis of chemicals was determined by the LDH cytotoxicity assay kit (MCE #HY‐K1090), followed by the manufacturer's instructions. Absorbance at 490 nm was measured by a Microplate Reader.

### 4.18 Public Datasets Analysis

We downloaded publicly available H3K27ac ChIP‐seq data from the ENCODE (https://www.encodeproject.org/) and from GSE116356 [17] for pNEN. Bulk RNA‐seq data for normal endocrine pancreas and pNEN were similarly downloaded from GSE156109 [53] and GSE116356, respectively. scRNA‐seq data were acquired from GSE159556 [54] for normal endocrine pancreas and from GSE162708 [7]. The ICGC dataset was acquired from https://dcc.icgc.org/releases/current/Projects.

For H3K27ac ChIP‐seq data analysis, files with bigwig format were downloaded and converted to bedGraph by the “bigWigToBedGraph” command with default parameters. Differential H3K27ac peaks were defined as the log_2_(peak intensity) > 3, H3K27ac DPGs were identified by overlapping differential peaks and genomic position of hg19 using the “bedtools‐intersect” command.

For bulk RNA‐seq data analysis, counts were downloaded, and the up‐regulated differential expression genes (up‐DEGs) were calculated by the “DESeq2” package with log_2_(FoldChange) > 2 and adjusted p value < 0.05.

For the ICGC dataset analysis, expression profiles (counts) from the ICGC‐PAEN‐AU cohort and corresponding clinical information were downloaded and analyzed. Gene expression levels were calculated and normalized to fragments per kilobase of transcript per million reads (FPKM) for downstream analysis.

For scRNA‐seq analysis, filtered matrices were acquired and integrated using the “Seurat” and “Harmony” packages. Cluster annotation was performed both automatically with the “SingleR” package and manually. Deconvolution analysis was performed by the “BayesPrism” package, using the ICGC dataset as bulk input with default parameters. The “Limma” package was used to calculate DEGs of tumor cell‐specific expression with an absolute value of log_2_(FoldChange) > 1 and adjusted p value < 0.05.

### 4.19 Statistics

GraphPad Prism (9.3.1) and R (4.2.3) were used to perform statistical analysis. All experiments were performed with at least 3 independent repeats. The statistical data of continuous variables in this study were given as mean ± standard deviation (SD) and analyzed by Student's *t*‐test between two groups or one‐way ANOVA with Tukey's multiple comparisons test between multiple groups. The Mann‐Whitney U test was used when the normality (Shapiro‐Wilk) test failed. Categorical variables were presented as frequencies or percentages and analyzed using the chi‐square test. Correlations were analyzed using the Spearman Correlation test. Survival analysis was performed using the log‐rank test. Statistical significance was determined as *p* < 0.05. Throughout all figures: ^ns^ *p* > 0.05, ^*^ *p* < 0.05, ^**^ *p* < 0.01, ^***^ *p* < 0.001, ^****^ *p* < 0.0001.

### 4.20 Study Approval

All experimental procedures involving human samples and clinical data have been approved by the Committee in The First Affiliated Hospital of Sun Yat‐Sen University (IIT‐2025‐045). Written informed consent was obtained from all patients before the collection of the sample and clinical data. All procedures were performed in compliance with relevant laws and institutional guidelines. All experimental procedures involving mice have been approved by the Committee in The First Affiliated Hospital of Sun Yat‐Sen University (2025‐029).

X.Y. conceived and designed the study. Y.Y. performed experiments and analyzed the results. Y.Y., J.X., Y.S., X.H., J.Z., E.Z., Z.Z., M.M., J.Y., and Z.L. assisted in the collection and analysis of clinical samples. X.Y. and Q.X. supervised and guaranteed the study. Y.Y., Q.X., and X.Y. prepared and reviewed the manuscript. All authors approved the final version of the manuscript.

## Conflicts of Interest

The authors declare no conflicts of interest.

## Supporting information




**Supporting File 1**: advs74162‐sup‐0001‐SuppMat.docx.

**Supporting File 2**: advs74162‐sup‐0002‐Data.zip.

## Data Availability

Any additional information required to reanalyze the data reported in this work paper is available from the lead contact upon reasonable request.
