# Supplementary material for: Calcium Channel Blockers Inhibit Pancreatic Neuroendocrine Neoplasms Progression via Cav1.2‐Epigenetic Circuit
Source: Adv Sci (Weinh). 2026 Feb 4;13(20):e16733. doi: 10.1002/advs.202516733 (PMC13067810; doi:10.1002/advs.202516733)
Supplement: Supplementary file 1 — Supporting File 1: advs74162‐sup‐0001‐SuppMat.docx. [file ADVS-13-e16733-s002.docx]

**Calcium Channel Blockers Inhibit Pancreatic Neuroendocrine Neoplasms Progression via Cav1.2-Epigenetic Circuit**

Yangyinhui Yu^1,3^, Qiongcong Xu^1,3^, Jinzhao Xie^1,3^, Mingjian Ma^1^, Xitai Huang^1^, Yinhao Shi^1^, Jiawei Zhou^1^, Enliang Zhu^1^, Ziyi Zhao^1^, Ning Zhang^2^, Zhide Liu^1^, Jingyuan Ye^1^ and Xiaoyu Yin^1,4^

^1^Department of Pancreato-Biliary Surgery, the First Affiliated Hospital of Sun Yat-Sen University, Guangzhou, 510080, China

^2^Department of Gastroenterology & Hepatology, the First Affiliated Hospital of Sun Yat-Sen University, Guangzhou, 510080, China
^3^These authors contributes equally

^4^Lead contact

**Supplemental Figures**

**
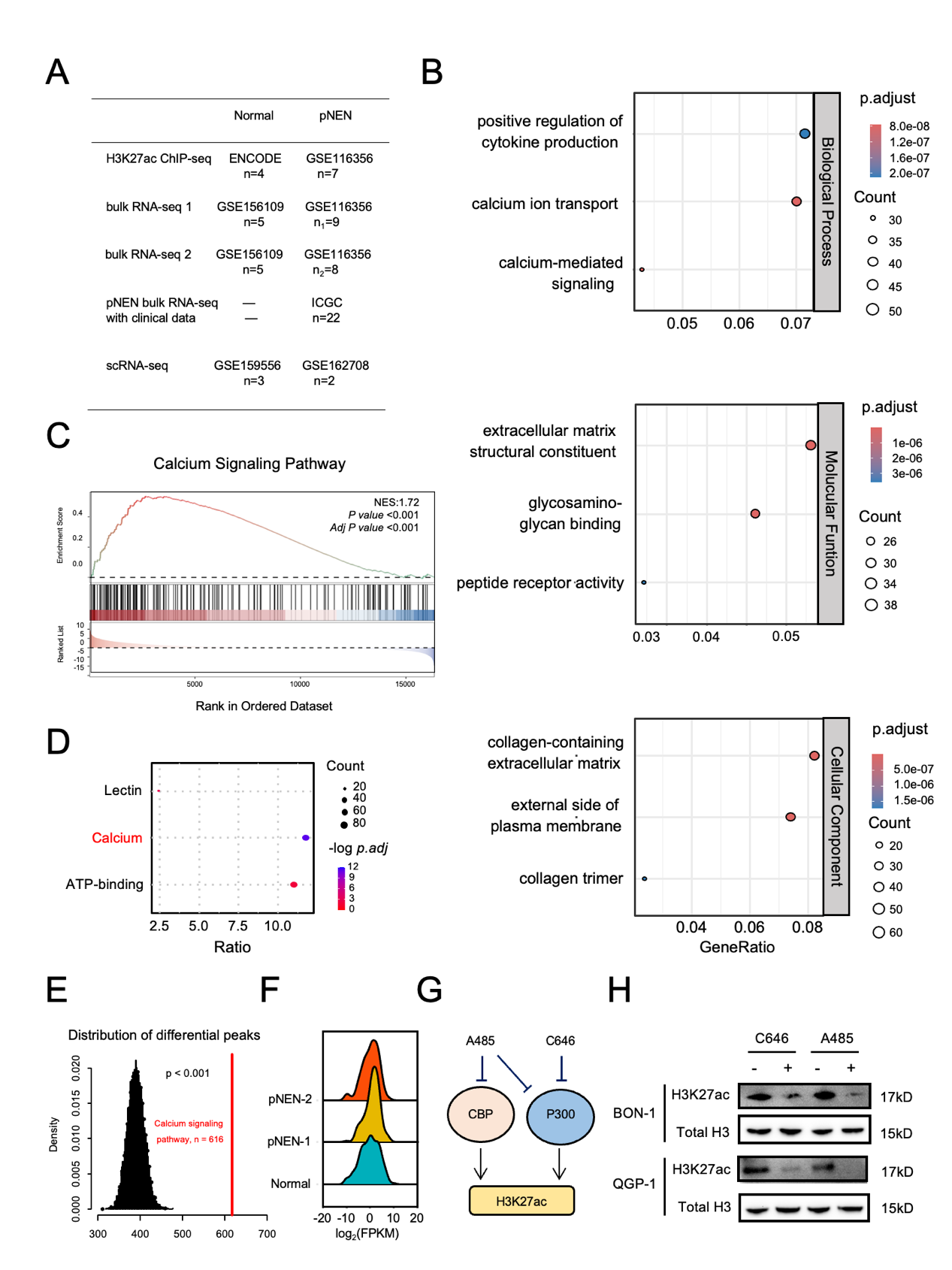
**

**Figure S1. Functional enrichment of H3K27 acetylation activated genes.**

(A) Table of multi-omic dataset included for analysis.

(B) GO analysis including biological process (BP), molecular function (MF) and cellular component (CC) in indicated gene set. Top 3 enriched terms were shown.

(C) GSEA analysis showing the enrichment of gene set in calcium signaling pathway.

(D) Ligand-receptor analysis of indicated gene set.
(E) Histogram showing the differential peaks distribution of H3K27ac genome-wide (black) and in calcium signaling pathway related gene locus (red).

(F) Ridges plot showing the expression level (log_2_FPKM) among calcium signaling pathway related genes in normal and two pNEN groups.

(G) Schematic illustrating the inhibitory target of A485 and C646. A485 inhibits P300/CBP complex and C646 specifically inhibits P300. KEGG enrichment analysis of overlapped genes. Calcium signaling pathway was identified as the most significantly enriched pathways. This suggests that this pathway may play crucial roles in pNEN progression.

(H) Western Blots showing reduced level of global H3K27ac after A485 and C646 treatment in BON-1 and QGP-1 cells. Total H3 was loaded as control.

**
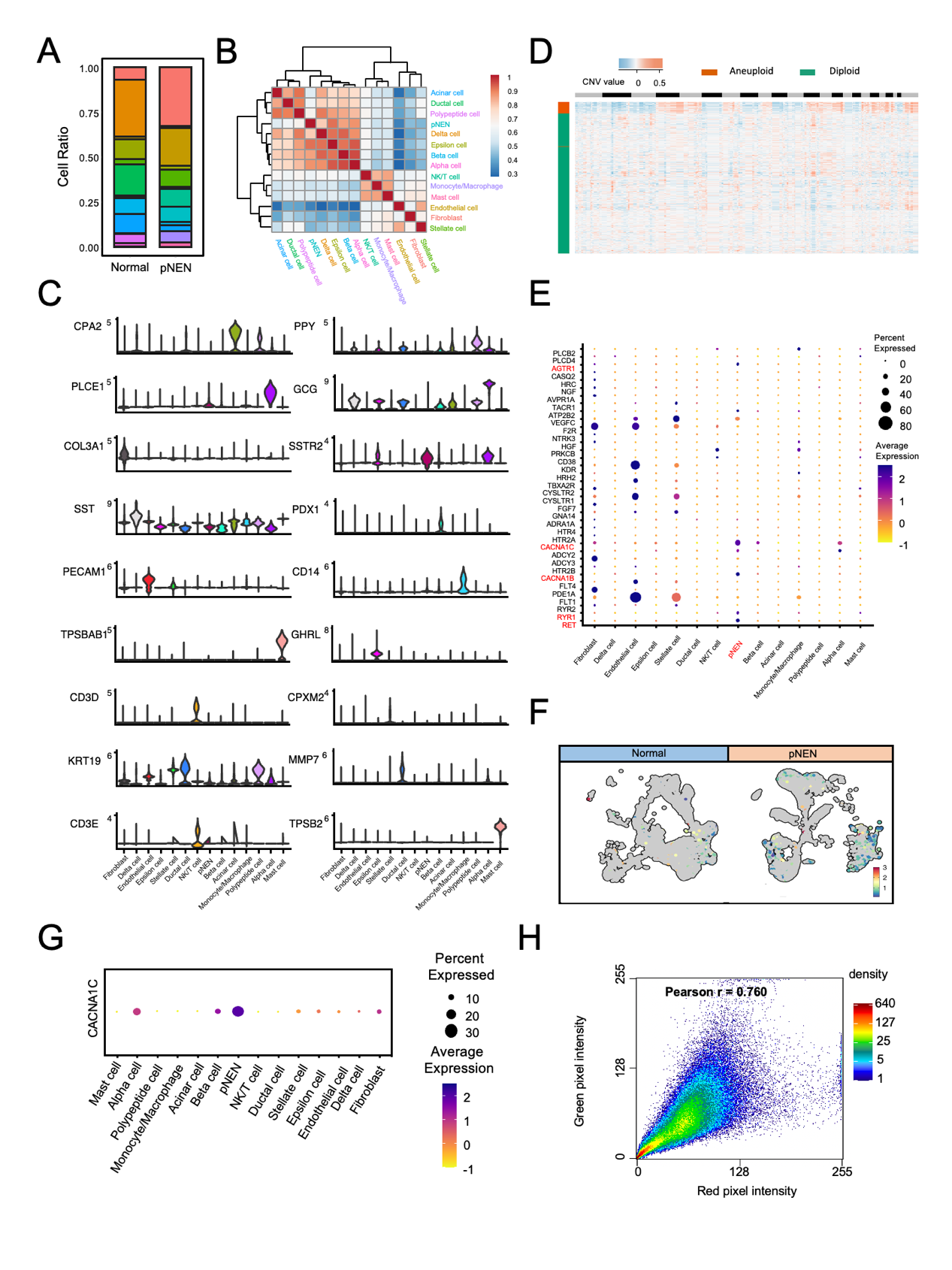
**

**Figure S2. Single-cell transcriptomic analysis of pNEN.**

(A) Cumulative bar plot showing proportions of each cell clusters.

(B) Clustered heatmap of cell-type specificity of each cell clusters.

(C) Violin plot showing expression of marker genes in each cell clusters.
(D) Heatmap of per-chromosomal CNV calculated in each cell. Red bar indicating the aneuploid (tumor) cells were identified.
(E) Dot plot showing expression level and percentage of gene expression in calcium signaling pathway related genes.
(F) UMAP presentation of *CACNA1C* expression separated in normal endocrine pancreas and pNEN group.
(G) Dot plot showing the *CACNA1C* expression preference among all cell types.

(H) Density plot showing positive correlations (Pearson r = 0.760) between red and green pixel intensity.


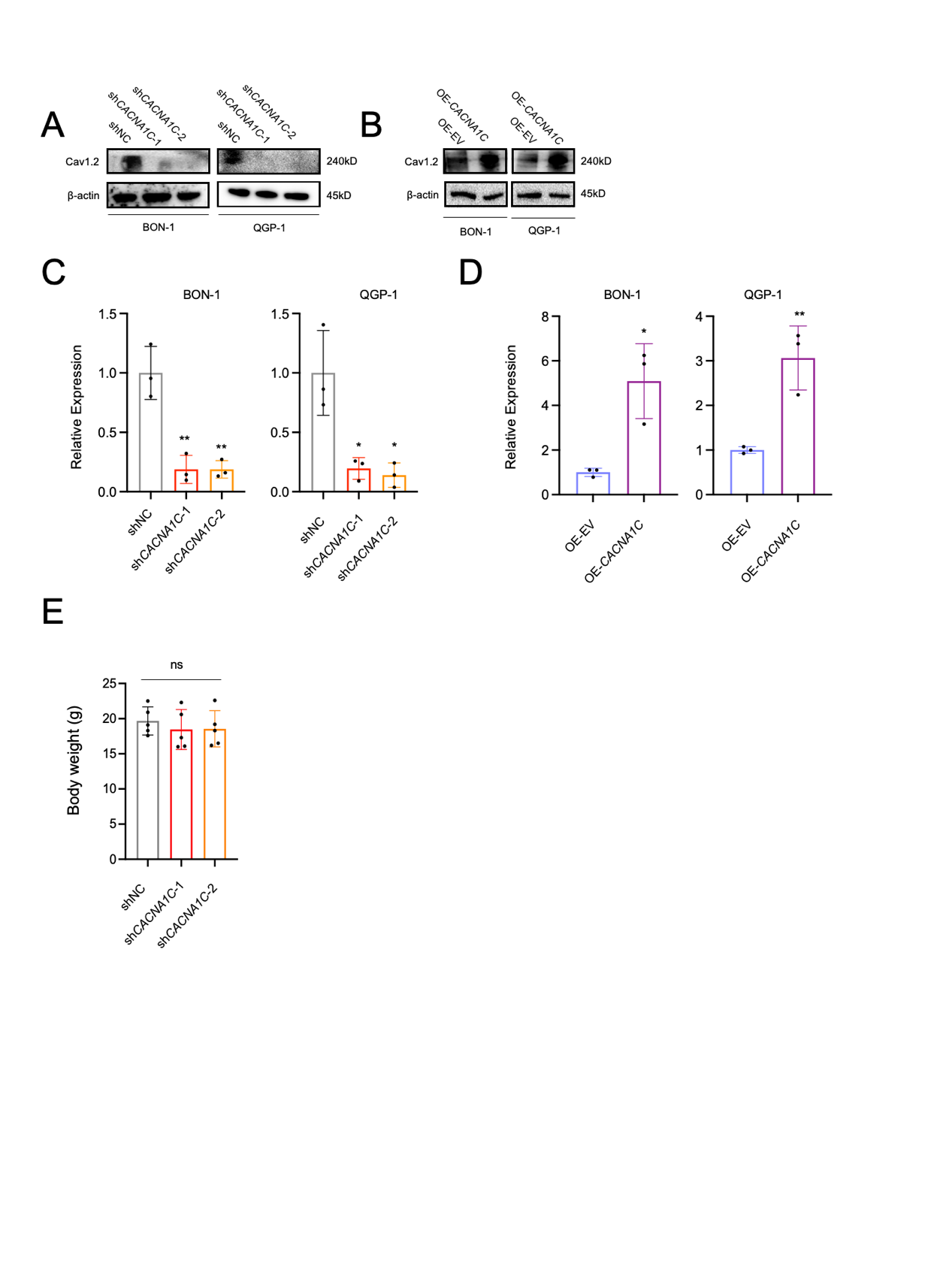


**Figure S3. Functional analysis of Cav1.2 to pNEN progression.**

(A) Western blots showing alteration of Cav1.2 in protein level between stable knockdown and control group.

(B) Western blots showing alteration of Cav1.2 in protein level between stable over-expression and control group.

(C) qRT-PCR of *CACNA1C* mRNA level relevant to A in BON-1 and QGP-1 cells. *p < 0.05; **p < 0.01 according to one-way ANOVA with Tukey’s multiple comparisons tests. Data was shown as mean ± SD.

(D) qRT-PCR of *CACNA1C* mRNA level relevant to B in BON-1 and QGP-1 cells. *p < 0.05; **p < 0.01 according to one-way ANOVA with Tukey’s multiple comparisons tests. Data was shown as mean ± SD.

(E) Body weight of mice inoculated with BON-1 cells among indicated groups. ns (not significant) according to one-way ANOVA with Tukey’s multiple comparisons tests. Sample size n = 5.

**
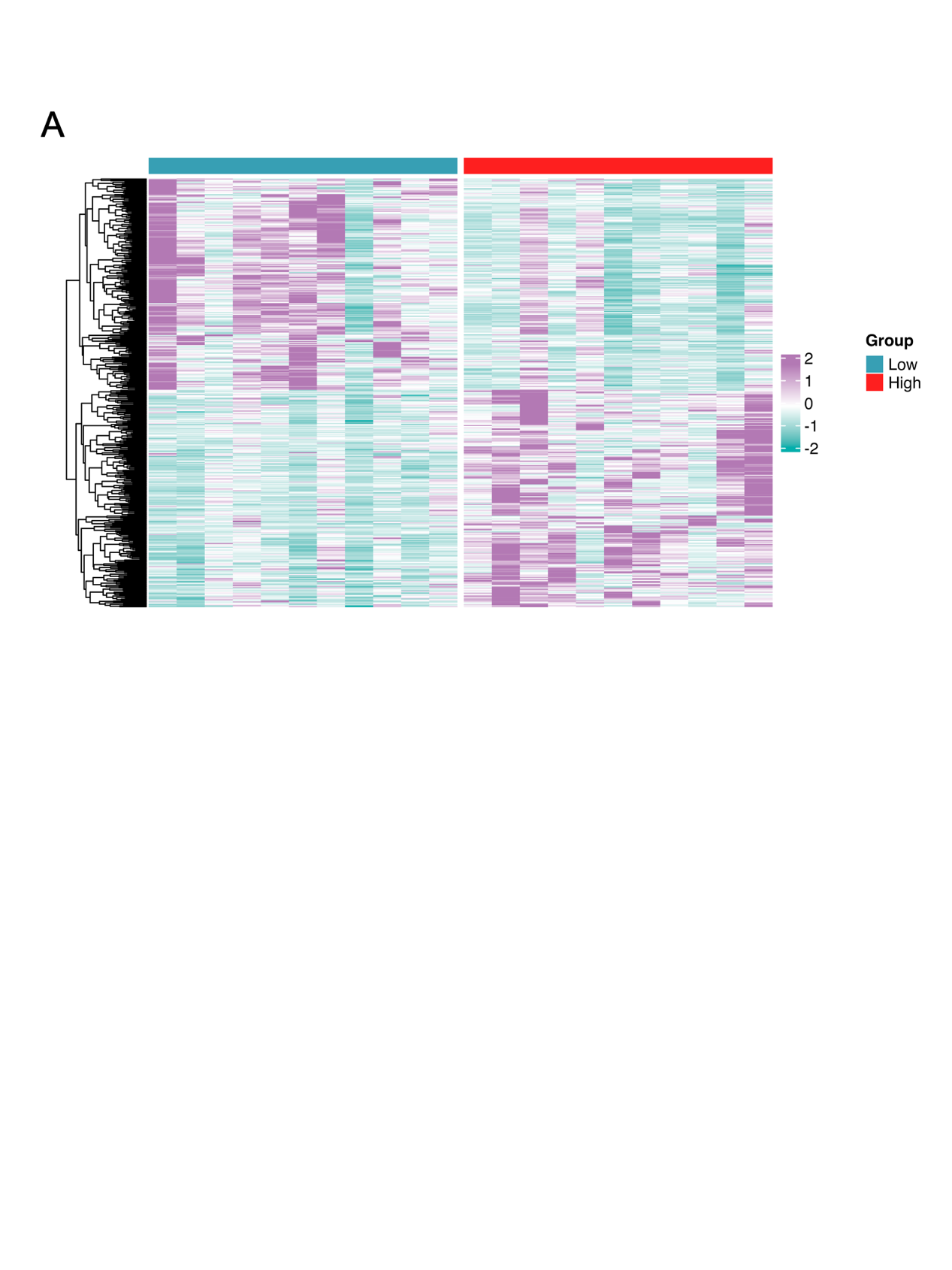

Figure S4. Correlations of Cav1.2 expression with malignant biological behaviors**

(A) Heatmap of DEGs among *CACNA1C* high and low group showing distinct expression profile.


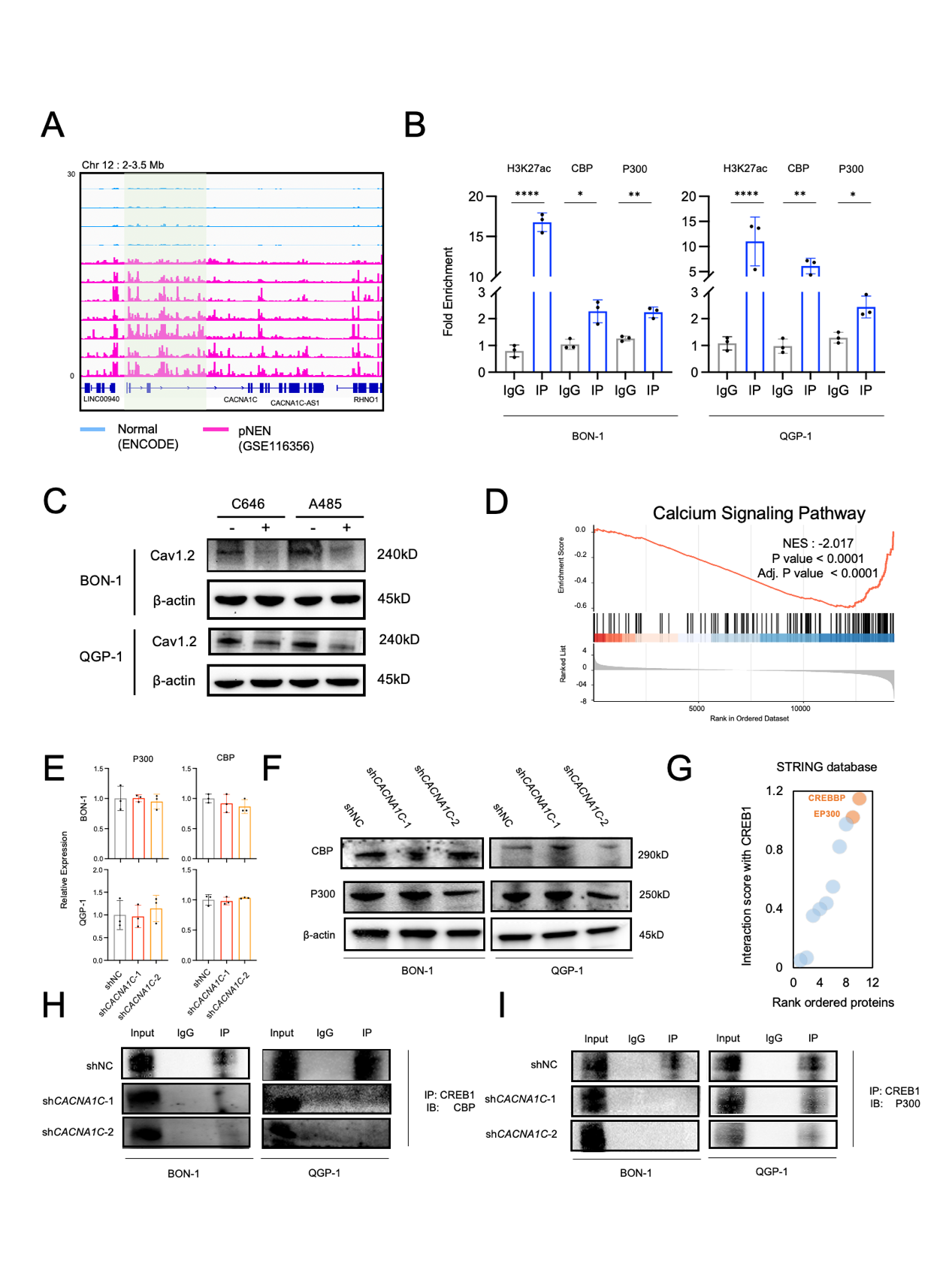


**Figure S5. Positive regulatory circuit of Cav1.2 with H3K27ac and P300/CBP**

(A) Track plots showing H3K27ac peaks in *CACNA1C* gene locus (ENCODE and GSE116356).

(B) ChIP-qPCR showing enrichment of H3K27ac, P300/CBP at *CANCA1C* locus. *p < 0.05; **p < 0.01; ****p < 0.0001 according to unpaired two-tailed Student’s t-test. Data was shown as mean ± SD.

(C) Western blots of Cav1.2 protein level after A485 or C646 treatment.

(D) GSEA analysis of down regulated genes comparing knockdown with control group.

(E) qRT-PCR of E*P300* and *CREBBP* mRNA level in shNC or two knockdown groups. ns (not significant) according to one-way ANOVA with Tukey’s multiple comparisons tests. Data was shown as mean ± SD.

(F) Western blots showing no difference of P300 and CBP in protein level after *CACNA1C* knockdown.

(G) Interaction scores with CREB1 from STRING database. Top two interacting candidates CBP and P300 were highlighted.

(H-I) co-IP analysis in which proteins were precipitated by CREB1 and blotted by CBP (H) and P300 (I).

**
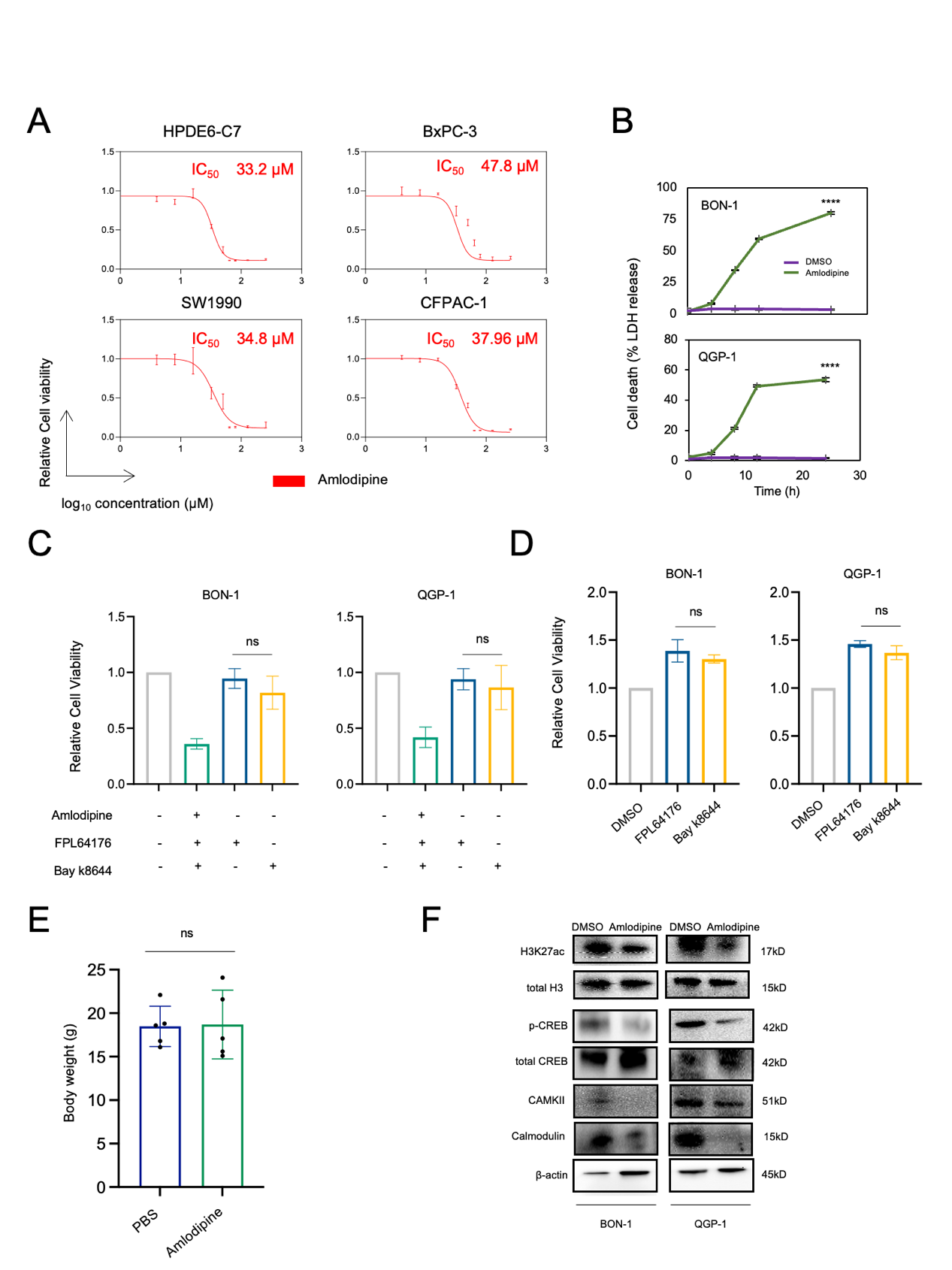
**

**Figure S6. Inhibitory effects of CCB in pNEN progression.**

(A) IC_50_ of amlodipine measured in normal pancreatic ductal epithelial cell (HPDE6-C7) and pancreatic ductal adenocarcinoma cell (BxPC-3, SW1990 and CFPAC-1).

(B) Percentage of LDH release (cytotoxicity) in different time series of amlodipine treatment. ****p < 0.0001 according to unpaired two-tailed Student’s t-test. Data was shown as mean ± SD.

(C) Rescue efficacy between Bay k8644 and FPL64176 in BON-1 and QGP-1 cells after amlodipine treatment. ns (not significant) according to one-way ANOVA with Tukey’s multiple comparisons tests Data was shown as mean ± SD.

(D) Pro-proliferative efficacy between Bay k8644 and FPL64176 in BON-1 and QGP-1 cells. ns (not significant) according to one-way ANOVA with Tukey’s multiple comparisons tests Data was shown as mean ± SD.

(E) Body weight of mice inoculated with BON-1 CDXs among PBS and amlodipine group. Data was shown as mean ± SD. Sample size n = 5.

(F) Western Blots of total H3 and H3K27ac, CaM, CAMKII and phosphorylated in DMSO and amlodipine group both in BON-1 and QGP-1 cells.

**
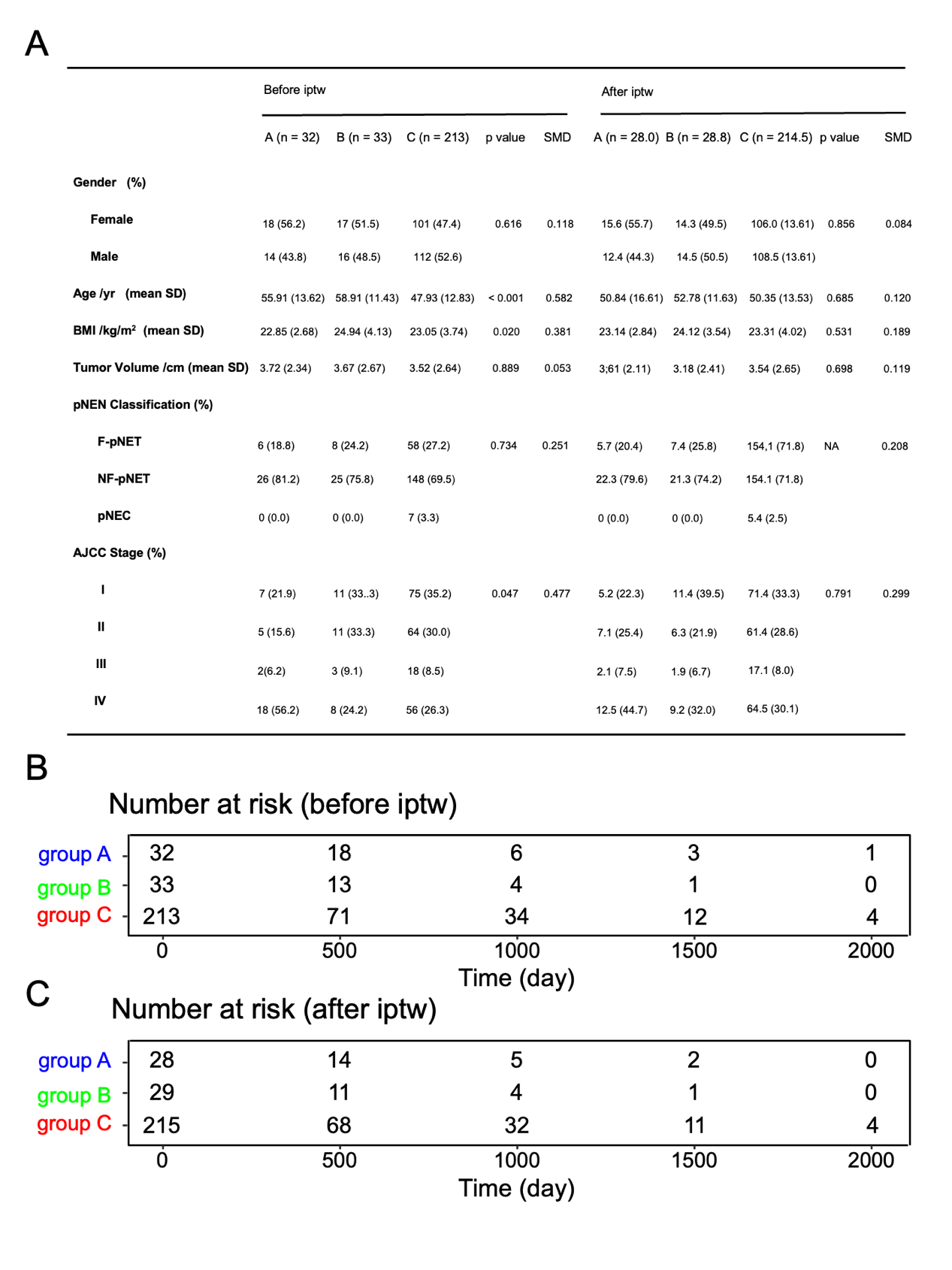
**

**Figure S7. Clinical data analysis of pNEN cohort.**

(A) Baseline characteristics of cohort before and after IPTW balancing.

(B-C) Numbers at risk among indicated groups before and after IPTW balancing.

**Supplemental Tables**

**Table S1** Reagents used in this study.

| **Reagents** | **Source** | **Identifier** |
| --- | --- | --- |
| DMEM | Gibco | Cat# 11965092 |
| DMEM/F12 | Gibco | Cat# C11330500BT |
| RPMI 1640 | Gibco | Cat# 11875093 |
| FBS | Cellcook | Cat# CM1002L |
| penicillin/streptomycin | ThermoFisher Scientific | Cat# 15140122 |
| PBS | Cellcook | Cat# CM2018 |
| VeZol | Vazyme | Cat# R411-01 |
| EndoFree Plasmid Midi Kit | TIANGEN | Cat# DP108 |
| ChamQ Universal SYBR qPCR Master Mix | Vazyme | Cat# Q711-02 |
| HiScript II Q RT SuperMix for qPCR | Vazyme | Cat# R223-01 |
| DAB for IHC | ServiceBio | Cat# G1212 |
| PHENOL CHLOROFORM pH 6.7/8.0 | Amresco | Cat# 0883-400ML |
| Universal DNA Purification Kit | TIANGEN | Cat# DP214 |
| SDS-PAGE gel | ACE | Cat# P0012A |
| Fura-2AM | Solarbio | Cat# IF1510 |
| Pluronic F-127 | Solarbio | Cat# P6790 |
| Matrigel | MCE | Cat# HY-K6005 |
| RIPA lysis buffer | Beyotime | Cat# P0013 |
| PVDF membrane | Millipore | Cat# IPVH10100 |
| Cell Counting Kit-8 | MCE | Cat# HY-K0301 |
| SDS-PAGE gel | ACE | Cat #ET12008Gel |
| Formaldehyde | Sigma | Cat# F8775 |
| DAPI | MCE | Cat# HY-D0814 |
| MNase | ThermoFisher Scientific | Cat #EN0181 |
| Protein A/G Magnetic Beads | Vazyme | Cat# PB101 |

**Table S2** Antibodies used in this study.

| **Antibodies** | **Source** | **Identifier** |
| --- | --- | --- |
| **Antibodies for WB/ChIP** | | |
| CACNA1C | Proteintech | Cat# 21774-1-AP |
| β-actin | Proteintech | Cat# 66009-1-Ig |
| H3K27ac | Active Motif | Cat# 39134 |
| Total H3 | Proteintech | Cat# 17168-1-AP |
| CAMKI | Santa Cruz | Cat# sc-137225 |
| CAMKII | Santa Cruz | Cat# sc-5306 |
| CAMKIV | Santa Cruz | Cat# sc-55501 |
| Calmodulin | Abclonal | Cat# A1185 |
| Total CREB1 | Proteintech | Cat# 12208-1-AP |
| P-CREB1 | Proteintech | Cat# 28792-1-AP |
| P300 | Santa Cruz | Cat# 54062 |
| CBP | Proteintech | Cat# 22277-1-AP |
| HRP-conjugated Goat Anti-Rabbit IgG (H+L) | Proteintech | Cat# SA00001-2 |
| HRP-conjugated Goat Anti-Mouse IgG (H+L) | Proteintech | Cat# SA00001-1 |
| **Antibodies for IF/IHC** | | |
| CACNA1C | Proteintech | Cat# 21774-1-AP |
| SSTR2 | Santa Cruz | Cat# sc-365502 |
| P300 | Santa Cruz | Cat# 54062 |
| CBP | Proteintech | Cat# 22277-1-AP |
| Goat Polyclonal anti-Rabbit Antibody (Alexa Fluor 647) | Abcam | Cat# Ab150079 |
| Goat Polyclonal anti-Mouse Antibody (Alexa Fluor 488) | Abcam | Cat# Ab6785 |
| Goat Anti-Rabbit IgG for IHC | ServiceBio | Cat# G1213 |
| Goat Anti-Mouse IgG for IHC | ServiceBio | Cat# G1212 |
| Normal Mouse IgG | Santa Cruz | Cat# 2729 |
| Normal Rabbit IgG | Santa Cruz | Cat# 68860 |

**Table S3** Softwares used in this study.

| **Software** | **Version** | **Source** |
| --- | --- | --- |
| R | 4.2.3 | https://www.r-project.org/ |
| Trim Galore | 4.1 | https://www.bioinformatics.babraham.ac.uk/projects/trim_galore |
| Fastqc | 0.11.9 | https://github.com/s-andrews/FastQC |
| Samtools | 1.3.1 | https://anaconda.org/bioconda/samtools |
| Subread | 2.0.1 | https://anaconda.org/bioconda/subread |
| Macs2 | 2.2.7.1 | https://anaconda.org/bioconda/macs2 |
| Hisat2 | 2.2.1 | https://anaconda.org/bioconda/hisat2 |
| DESeq2 | 1.40.2 | https://www.bioconductor.org/packages/release/bioc/html/DESeq2.html |
| Bowtie2 | 2.5.0 | https://anaconda.org/bioconda/bowtie2 |
| Limma | 3.56.2 | https://www.bioconductor.org/packages/release/bioc/html/limma.html |
| Deeptools | 3.5.1 | https://anaconda.org/bioconda/deeptools |
| ClusterProfiler | 4.8.3 | https://www.bioconductor.org/packages/release/bioc/html/clusterProfiler.html |
| Seurat | 5.0.1 | https://github.com/satijalab/seurat |
| scCustomize | 1.1.3 | ﻿https://github.com/samuel-marsh/scCustomize |
| Copykat | 1.1.0 | https://github.com/navinlabcode/copykat |
| BayesPrism | 2.2.2 | https://github.com/Danko-Lab/BayesPrism |
| Graphpad Prism | 9.3.1 | www.graphpad.com |

**Table S4** Oligonucleotides used in this study.

| **Oligonucleotides** | **Source** | **Identifier** |
| --- | --- | --- |
| *CACNA1C*-Forward (5’-3’)  CGTTCTCATCCTGCTCAACA | This study | N/A |
| *CACNA1C*-Reverse (5’-3’)  TATGCTCCCAATGACGATGA | This study | N/A |
| *GAPDH*-Forward (5’-3’)  GGAGCGAGATCCCTCCAAAAT | This study | N/A |
| *GAPDH*-Reverse (5’-3’)  GGCTGTTGTCATACTTCTCATGG | This study | N/A |
| ChIP-*CACNA1C*-Forward (5’-3’)  CCACGGCTTCCTCGAATCTT | This study | N/A |
| ChIP-*CACNA1C*-Reverse (5’-3’)  GGTCCAGCCTTACCTTGGTG | This study | N/A |
| ChIP-NC-Forward (5’-3’)  GGCATTAACTCCCCTGTGCT | This study | N/A |
| ChIP-NC-Reverse (5’-3’)  GTTTTCACCAGTGTCGTGGC | This study | N/A |
| sh*CACNA1C*-1 (5’-3’)  GGGTTGTCTGTGTGCATATGT | This study | N/A |
| sh*CACNA1C*-2 (5’-3’)  GCCTTCTTCATGATGAACATC | This study | N/A |

**Table S5.** Tumor cell specific expression matrix after deconvolution analysis, Related to Figure 4.

**Table S6.** Differential expression genes summary, Related to Figure 5.
